# Antigenic cartography using variant-specific hamster sera reveals substantial antigenic variation among Omicron subvariants

**DOI:** 10.1073/pnas.2310917121

**Published:** 2024-07-30

**Authors:** Barbara Mühlemann, Jakob Trimpert, Felix Walper, Marie L. Schmidt, Jenny Jansen, Simon Schroeder, Lara M. Jeworowski, Jörn Beheim-Schwarzbach, Tobias Bleicker, Daniela Niemeyer, Anja Richter, Julia M. Adler, Ricardo Martin Vidal, Christine Langner, Daria Vladimirova, Samuel H. Wilks, Derek J. Smith, Mathias Voß, Lea Paltzow, Christina Martínez Christophersen, Ruben Rose, Andi Krumbholz, Terry C. Jones, Victor M. Corman, Christian Drosten

**Affiliations:** ^a^Institute of Virology, Charité - Universitätsmedizin Berlin, corporate member of Freie Universität Berlin, Humboldt-Universität zu Berlin, and Berlin Institute of Health, Berlin 10117, Germany; ^b^German Centre for Infection Research (Deutsches Zentrum für Infektionsforschung), Berlin 10117, Germany; ^c^Institut für Virologie, Freie Universität Berlin, Berlin 14163, Germany; ^d^Center for Pathogen Evolution, Department of Zoology, University of Cambridge, Cambridge CB2 3EJ, United Kingdom; ^e^Institute for Infection Medicine, Christian-Albrechts-Universität zu Kiel and University Medical Center Schleswig-Holstein, Kiel 24105, Germany; ^f^Labor Dr. Krause und Kollegen Medizinisches Versorgungszentrum GmbH, Kiel 24106, Germany; ^g^Labor Berlin–Charité Vivantes GmbH, Berlin 13353, Germany

**Keywords:** SARS-CoV-2, vaccines, immunology

## Abstract

Severe acute respiratory syndrome Coronavirus 2 (SARS-CoV-2) has developed substantial antigenic variability. As the majority of the population now has pre-existing immunity due to infection or vaccination, the use of experimentally generated animal immune sera can be valuable for measuring antigenic differences between virus variants. Here, we immunized Syrian hamsters by two successive infections with one of nine SARS-CoV-2 variants. Their sera were titrated against 16 SARS-CoV-2 variants, and the resulting titers were visualized using antigenic cartography. The antigenic map shows a condensed cluster containing all pre-Omicron variants (D614G, Alpha, Delta, Beta, Mu, and an engineered B.1+E484K variant) and considerably more diversity among a selected panel of Omicron subvariants (BA.1, BA.2, BA.4/BA.5, the BA.5 descendants BF.7 and BQ.1.18, the BA.2.75 descendant BN.1.3.1, the BA.2-derived recombinants XBB.2 and EG.5.1, and the BA.2.86 descendant JN.1). Some Omicron subvariants were as antigenically distinct from each other as the wildtype is from the Omicron BA.1 variant. Compared to titers measured in human sera, titers in hamster sera are of higher magnitude, show less fold change, and result in a more compact antigenic map topology. The results highlight the potential of sera from hamsters for the continued antigenic characterization of SARS-CoV-2.

## Significance

Since late 2020, severe acute respiratory syndrome Coronavirus 2 (SARS-CoV-2) variants capable of evading the immune response from previous infection or vaccination have emerged. Understanding and tracking changes in antigenicity is a crucial component of vaccine design and update. Finding human first-infection sera to measure antigenic distances between novel variants is becoming impossible, as most people have been infected or vaccinated. Therefore, an alternative (nonhuman) model is needed. We evaluated hamster sera as a surrogate to human first-infection sera to measure antigenic differences between recently circulating variants. We find a closer antigenic relationship between the pre-Omicron variants circulating in 2020 and 2021 and larger distances between Omicron variants.

The severe acute respiratory syndrome Coronavirus 2 (SARS-CoV-2) pandemic has caused over 774 million infections and over 7 million deaths as of January 2024 (1). The provision of vaccines greatly reduced infection fatality rates and disease burden (2). However, antigenic variability of SARS-CoV-2, facilitated by substitutions in the viral spike protein, resulted in the evasion of antibody neutralization induced by infection or vaccination (3–6). In particular, SARS-CoV-2 variant of concern (VOC) Omicron and its subvariants arising from late 2021 show strong escape from serum neutralization in individuals infected with pre-Omicron variants such as the initial variant (spike D614G), and variants Alpha, Beta, Gamma, and Delta (7–13).

In late 2022, the circulating viruses were dominated by Omicron subvariants with convergent substitutions at several spike amino acid positions (346, 444 to 446, 450, 452, 460, 486, 490, and 493). These included the BA.5-derived variants BF.7 and BQ.1.18, which respectively acquired one (R346T) or three (R346T, K444T, N460K) additional substitutions in the spike protein, as well as several BA.2.75-derived variants, including BN.1.3.1 with an additional 10 substitutions in the spike protein compared to BA.2. In 2023, circulation was dominated by variants of the recombinant XBB lineage which arose from the recombination of two BA.2 lineages, BJ.1 and BM.1.1.1 (14). These included the XBB.1.5, XBB.1.9, and XBB.1.16 variants. Various XBB-descendant variants convergently acquired substitutions at positions 356, 403, 453, 455, 456, 478, or 486 in mid-2023, resulting in the dominant circulation of variants such as EG.5.1 or HV.1 (14, 15). In August 2023, the BA.2-descendant BA.2.86 variant was first observed, with an additional 34 substitutions in the spike protein compared to BA.2, and in December of that year, the BA.2.86-descendant variant JN.1, with one additional substitution (L455S) compared to BA.2.86, became the dominant variant in many countries (https://www.who.int/docs/default-source/coronaviruse/21112023_ba.2.86_ire.pdf) (16, 17). The Omicron variants displayed strong immune escape compared to previously circulating variants (7–13, 16–22), which prompted the development and licensing of bivalent vaccine formulations containing additional antigens based on either the Omicron BA.1 or BA.4/BA.5 subvariants in summer 2022 (23, 24) and a further update of the vaccine to a monovalent formulation based on the XBB.1.5 variant a year later (https://www.fda.gov/vaccines-blood-biologics/updated-covid-19-vaccines-use-united-states-beginning-fall-2023) (25). Ongoing monitoring of the antigenic properties of circulating variants is critical for determining the need for future updates of vaccine antigens.

In initial attempts to track SARS-CoV-2 antigenic evolution, human primary infection or vaccination sera were used to investigate antigenic differences between SARS-CoV-2 variants by cross-titration using different serum neutralization tests (3–5, 7–9, 11, 13, 26, 27). The use of primary infection sera allows the measurement of the base antigenic distances between variants, i.e., using serum neutralization titers that are not confounded by previous infections or vaccinations. Results from cross-titrations can be visualized using antigenic cartography to show antigenic relationships between variants (28), as routinely used to aid the selection of vaccine strains for the human seasonal influenza vaccine. Sera from individuals exposed to multiple variants may lead to an underestimation of antigenic distances (29, 30) when included in an antigenic map. However, data from multiexposure sera can be added as a third dimension to a two-dimensional antigenic map, to create an “antibody landscape”, which provides a visualization of the antigenic space that an individual has reactivity to (29). With the exception of young children, large fractions of the population have now been infected or vaccinated (31–33). Therefore, obtaining sera from subjects with primary infections with new variants is becoming very difficult. For example, while it was still possible to identify unvaccinated individuals with first infections with Omicron BA.1 and BA.2 (8, 9, 13), doing so for Omicron BA.5 and future variants will be difficult. Likewise, identifying individuals with known infection histories (e.g., vaccinated with a breakthrough infection with a known variant) will also become more challenging as testing programs are discontinued.

An alternative is to use sera from experimental animals with serum antibody response characteristics similar to that of humans. Using experimental animal infections allows the generation of sera for any variant, with predefined infection histories, taken at standardized times after infection (30). Further, due to the higher titers resulting from experimental infections, animal sera could provide more “dynamic range’’ as titers will not become undetectable as readily as in human convalescent sera when tested against variants with large antigenic distance (11, 34). Syrian hamsters are a widely used animal model for COVID-19. Upon infection with SARS-CoV-2, these animals develop mild to moderate disease and show seroconversion (34–37). However, there is still a lack of experience with standardization of infection and assay conditions, and only one laboratory has developed and presented a complete approach that includes strain selection, immunization scheme, and serum neutralization evaluation (34, 38).

Here, we provide an independent approach to Syrian hamster-based antigenic cartography. We use an efficient immunization scheme based on low- and subsequent high-dose intranasal inoculation and a refined neutralization assay criterion with titers determined on a continuous scale using a neutralization threshold of 90%. We characterize antigenic differences between SARS-CoV-2 variants circulating up to December 2023 using antigenic cartography (28). The results reveal considerable antigenic diversity among Omicron subvariants, which are often as antigenically distant from each other as BA.1 is from the pre-Omicron variants.

## Results

We infected 29 6-wk-old female Syrian hamsters with the D614G isolate B.1 (n = 3), Alpha (n = 2), Delta (n = 3), Beta (n = 3), an engineered B.1 virus containing the escape mutation E484K, termed B.1+E484K (n = 3), Omicron BA.1 (n = 3), Omicron BA.2 (two different isolates, BA.2 and BA.2-12, n = 3 each), Omicron BA.5 (n = 3), and Omicron XBB.2 (n = 3) (*SI Appendix*, Table S1). To increase serum neutralization capacity and in an attempt to reduce disease burden, hamsters were infected twice, using a low virus inoculum for the initial infection, followed by a high-dose re-infection 3 wk later. Sera were collected 2 wk after the second infection. To evaluate whether the reactivity patterns measured in the hamster sera reflect those of human sera, we also titrated sera from unvaccinated humans first infected with D614G (n = 51), Alpha (n = 22), and Beta (n = 40) against D614G, Alpha, Beta, and Delta in the same assay.

Sera were titrated by plaque reduction neutralization test (PRNT) on Vero E6 cells against live virus isolates of D614G, Alpha, Delta, Beta, Mu, B.1+E484K, and Omicron subvariants BA.1, BA.2 (two isolates, BA.2 and BA.2-12), BA.4, BA.5, BF.7, BQ.1.18, XBB.2, BN.1.3.1, EG.5.1, and JN.1 (*SI Appendix*, Table S1 and Datasets S1 and S2). The BF.7, BQ.1.18, and BN.1.3.1 variants were only titrated against the D614G, BA.1, BA.2, and BA.5 sera, and the XBB.2 variant was additionally titrated against the XBB.2 sera. The EG.5.1, and JN.1 variants were only titrated against the BA.1, BA.2, BA.5, and XBB.2 sera. The BA.5 sera were only titrated against the D614G and BA.1, BA.2, BA.5, BF.7, BQ.1.18, BN.1.3.1, and XBB.2 variants and the XBB.2 sera were only titrated against the D614G, BA.1, BA.5, XBB.2, EG.5.1, and JN.1 variants. All other variants and sera were titrated all-against-all.

### Titer Determination.

We investigated different methods for inferring titers from plaque counts (*SI Appendix*, *Titer Determination*). We tested the effects of using different neutralization criteria on the fold change and the antigenic map topology by inferring neutralization titers when 50, 75, 90, and 99% of all plaques were neutralized. Overall, the fold change estimated by the titers inferred with different neutralization criteria were comparable (*SI Appendix*, Figs. S1 and S2). When inferring antigenic maps for each of the different neutralization criteria (*SI Appendix*, Fig. S3), we found that in the maps from NT75 and NT90 titers, generally the Omicron BA.1 and BA.2 variants are positioned within the groups of BA.1 and BA.2 sera, respectively, whereas in the NT50 maps, these variants are positioned away from their homologous sera, suggesting that NT50 titers slightly overestimate the reactivity of those variants in relation to their homologous sera. We proceeded to use NT90 titers for the remaining analyses (*SI Appendix*, *Titer Determination*). In addition, we investigated whether discrete and continuous titers would allow us to draw similar conclusions with regard to measured fold change (*SI Appendix*, Fig. S4) and the inferred antigenic maps (*SI Appendix*, Fig. S5) and found fold changes and the antigenic maps to be similar between different methods (*SI Appendix*, Figs. S4 and S5). In the following analyses of the hamster sera, we therefore refer to NT90 titers as determined using the neutcurve package (version 0.5.7) (39), constraining the lower end of the neutralization curve to zero (*SI Appendix*, Figs. S1–S6, Tables S2 and S3, and *Titer Determination* and Dataset S2). For the human sera, titers were determined using a neutralization cutoff of 50%.

### Analysis of Raw Titers and Fold Change from Homologous Variant.

**Fig. 1.**
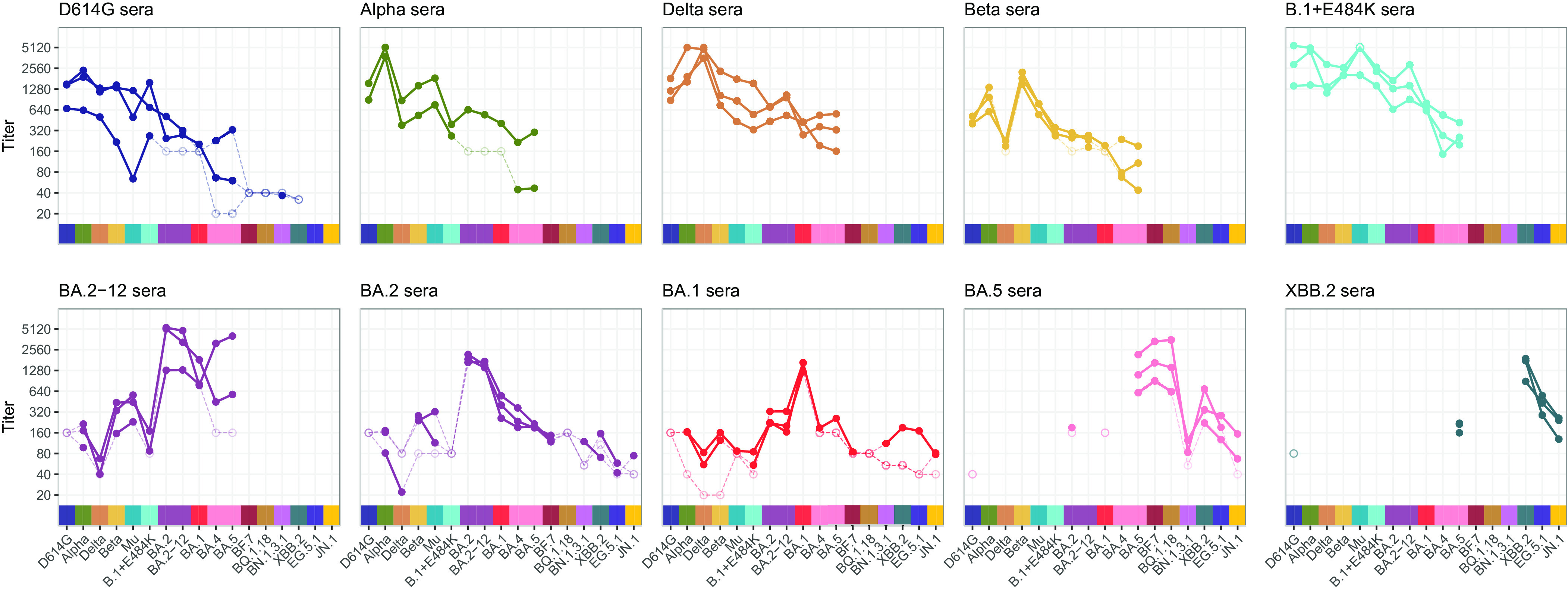
Neutralizing titers of 17 live virus isolates titrated against sera from hamsters twice infected with D614G, Alpha, Beta, Delta, Mu, B.1+E484K, Omicron BA.1, Omicron BA.2 (two isolates), Omicron BA.5, and Omicron XBB.2. Variants that were titrated are D614G, Alpha, Beta, Delta, Mu, B.1+E484K, Omicron BA.1, Omicron BA.2 (two isolates), Omicron BA.4, Omicron BA.5, Omicron BF.7, Omicron BQ.1.18, Omicron XBB.2, Omicron BN.1.3.1, Omicron EG.5.1, Omicron JN.1. Detectable titers are shown as filled in circles, nondetectable titers are shown as empty circles. Lines connecting nondetectable titers are dashed. Since not all variant and serum pairs were tested in all dilutions, nondetectable titers range from <1:160 to <1:20.

All animals generated high antibody titers, with the highest titers for each serum ranging from 666 to >5,120 (Fig. 1). Reactivity patterns among the hamsters infected with the same isolate were largely uniform. Titers between the two BA.2 isolates (*SI Appendix*, Fig. S7*A*) and between the BA.4 and BA.5 isolates (*SI Appendix*, Fig. S7*B*) were similar.

In general, sera raised by infection with pre-Omicron variants have at least some reactivity to each nonhomologous pre-Omicron variant, although the Beta sera have greatly reduced titers when titrated against the Delta variant (Fig. 1 and *SI Appendix*, Fig. S8). Against Omicron variants BA.1, BA.2, and BA.4/BA.5, titers of pre-Omicron sera tend to be lower, being highest against Omicron BA.2 and lowest against Omicron BA.4/BA.5. The D614G sera are not reactive or have low titers against Omicron variants BF.7, BQ.1.18, BN.1.3.1, and the XBB.2 recombinant. The sera raised by infection with Omicron variants show low or nondetectable titers against pre-Omicron variants. BA.1 and BA.2 sera retain some reactivity against BA.2 or BA.1, respectively, and have low or nondetectable titers against BF.7, BQ.1.18, BN.1.3.1, XBB.2, EG.5.1, and JN.1 Omicron variants. The BA.5 sera have high titers against the related BA.5, BF.7, and BQ.1.18 variants and, surprisingly, also show relatively high reactivity against the recombinant XBB.2 and EG.5.1, but not against BA.1, BA.2, BN.1.3.1, or JN.1. XBB.2 sera show high titer against the homologous XBB.2 variant, retain some reactivity against EG.5.1, BA.5, and JN.1, but show no reactivity against D614G.

The raw titers and fold change show the considerable antigenic distance between pre-Omicron and Omicron variants. The differences in titers measured against different Omicron variants in Omicron sera also indicate a large antigenic diversity among Omicron variants.

### Antigenic Cartography.

**Fig. 2.**
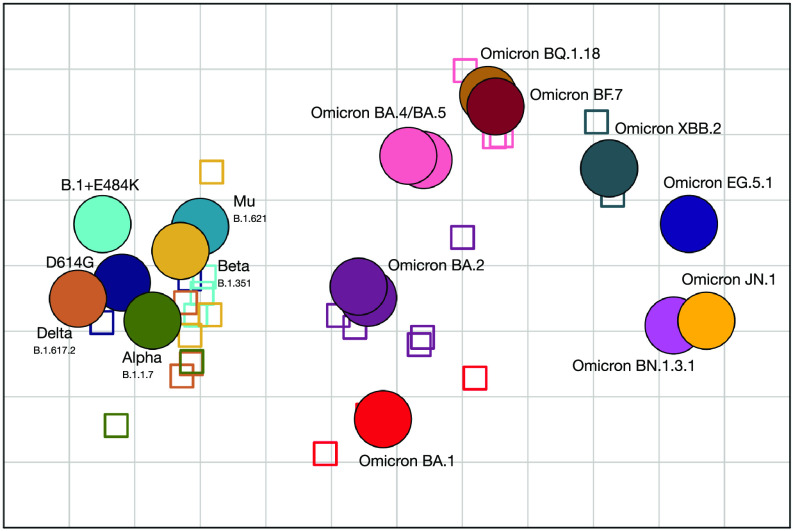
Antigenic map showing antigenic relationships between SARS-CoV-2 variants and sera. Distances between each variant and serum correspond to the fold change to the maximum titer for each serum. Viruses are shown as circles, sera as squares, with sera colored by the color of their homologous variant (blue: D614G, green: Alpha, dark-yellow: Beta, orange: Delta, green-blue: Mu, cyan: B.1+E484K, red: BA.1, orchid: BA.2 (2×, on top of each other), pink: BA.4 and BA.5, ochre: BQ.1.18, maroon: BF.7, sea-green: XBB.2, light-orchid: BN.1.3.1, dark blue: EG.4.1, yellow: JN.1). The side length of each grid square corresponds to a twofold serum dilution in the neutralization assay. The rotation of the map is arbitrary and is here oriented to correspond to previously published maps (8, 34).

We used antigenic cartography (28), implemented in the Racmacs package (40), to visualize the antigenic relationships between SARS-CoV-2 variants and sera. In antigenic maps, the distance between each variant and a serum corresponds to the fold change compared to the variant with the highest titer against that serum. The antigenic map (Fig. 2) provided a good fit to the data and was robust to missing titer data and noise (*SI Appendix*, Figs. S9–S18 and *Assessing Antigenic Map Model Fit*).

The antigenic map (Fig. 2) of the titers presented in Fig. 1 shows a cluster consisting of pre-Omicron variants (D614G, Alpha, Delta, Beta, Mu, B.1+E484K), and a looser grouping of Omicron subvariants, consistent with the titer and fold change pattern observed in Fig. 1 and *SI Appendix*, Fig. S8. Many Omicron variants (e.g., BA.1 vs. BA.4/BA.5, BA.4/BA.5 vs. BN.1.3.1, and BA.4/BA.5 vs. EG.5.1 and JN.1) are as antigenically distant from each other as BA.1 is from the pre-Omicron variants. Within the Omicron subvariants, BA.4/BA.5, BF.7, and BQ.1.18 group relatively closely, in agreement with the descent of BF.7 and BQ.1.18 from BA.5. The map shows larger antigenic distances between the other Omicron variants than between the pre-Omicron variants. The JN.1 variant is positioned furthest from D614G and distant from the previously circulating XBB.2 and EG.5.1 variants. Interestingly, the map suggests a close antigenic similarity between BN.1.3.1 and JN.1. The larger distances in the antigenic map are mainly caused by the inclusion of titrations against the BA.1, BA.2, BA.5, and XBB.2 sera, because excluding those serum groups moves the Omicron variants closer together (*SI Appendix*, Fig. S15). This indicates that there are differences among Omicron variants that are only detected by Omicron sera and not by pre-Omicron sera.

To limit the future use of animals needed, we investigated the minimum number and type of sera required for appropriate antigenic map triangulation by subsampling the sera included in the map. For this analysis, we considered only those virus isolates and sera that had been included in all titrations, so that each variant and serum is positioned by the same number of titrations. Therefore, we excluded titrations between Omicron BA.5 and XBB.2 sera and BF.7, BQ.1.18, XBB.2, BN.1.3.1, EG.5.1, and JN.1 variants. We constructed antigenic maps from randomly subsampled combinations of sera and serum groups (*SI Appendix*, Figs. S15, S19, and S20). Both BA.1 and BA.2 serum groups are required for adequate positioning of the Omicron variants (*SI Appendix*, Figs. S15 and S20 *A*–*C*). To determine the number of pre-Omicron serum groups required for positioning the antigens and sera on the antigenic map, we subsampled from the pre-Omicron serum groups, always including the BA.1 and BA.2 sera. This resulted in antigenic maps that were largely similar to the map made from all sera (Fig. 2), even when only one pre-Omicron serum group was included alongside the BA.1 and BA.2 serum groups (*SI Appendix*, Figs. S19 and S20*C*). We further subsampled maps made from three to seven serum groups to contain only one or two sera per serum group (*SI Appendix*, Fig. S20 *D* and *E*) and found the antigen positions of those maps to be more different to the full map compared to the maps made from all sera within a serum group (*SI Appendix*, Fig. S20*C*). This suggests that, in this dataset, reducing the number of sera within a serum group negatively impacts antigenic map topology. However, the number of different pre-Omicron virus variant serum groups can be decreased.

### Comparison to Human Sera.

**Fig. 3.**
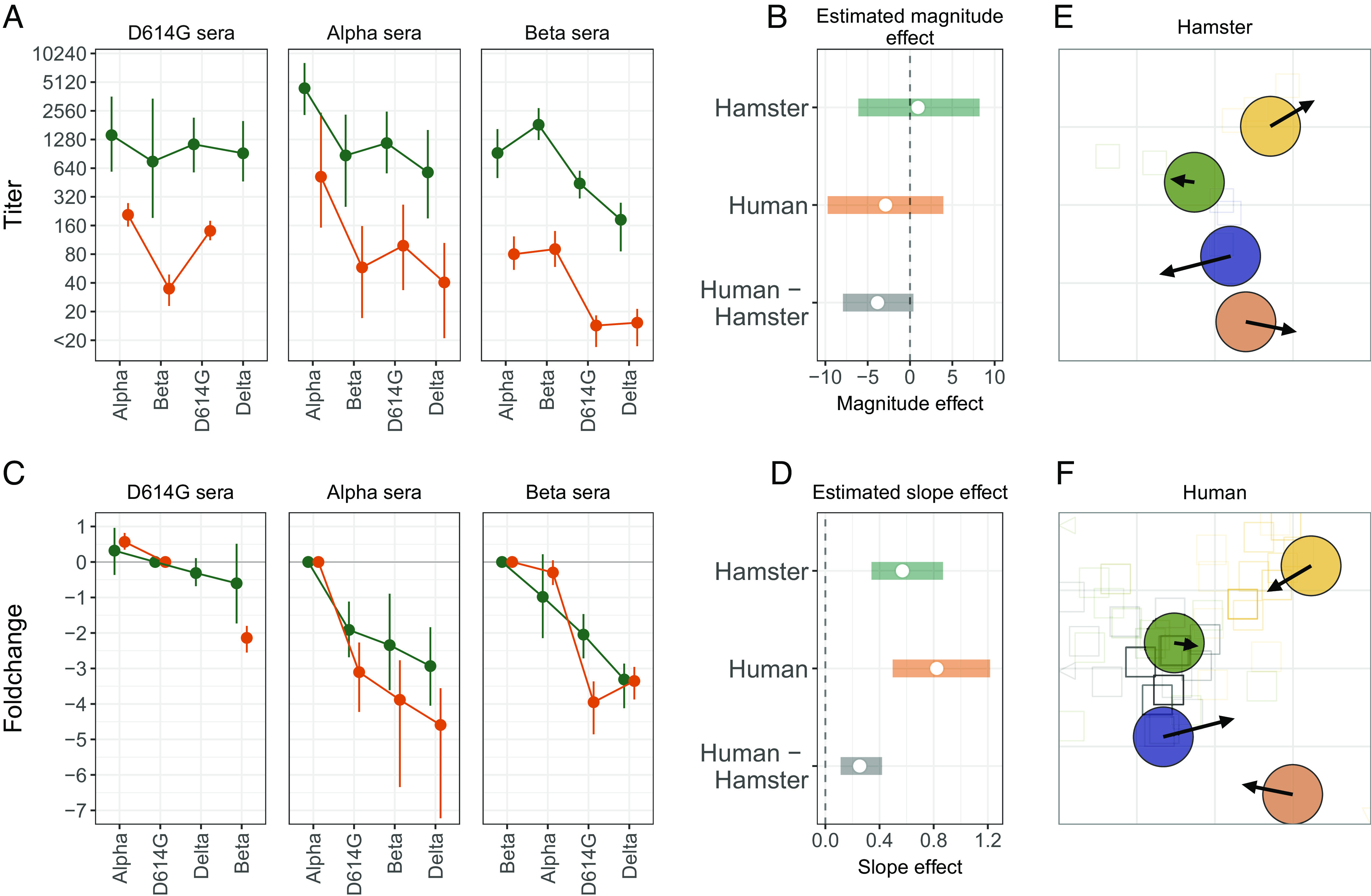
Comparison of hamster and human sera. Comparison is shown according to titer magnitude (*A* and *B*), fold change (*C* and *D*), and antigenic maps (*E* and *F*). (*A*) GMT of hamster (green) and human (red) sera. Dots correspond to the GMT, lines to the estimated 95% highest posterior density interval (HPDI). (*B*) Posterior distributions of the estimated titer magnitude explained by whether a titer was measured using hamster (green) or human (red) sera, as well as the contrast of these distributions (Human–Hamster). The dot corresponds to the mean, the bar to the 95% HPDI. (*C*) Fold change estimated in hamster (green) and human (red) sera. Variants are ordered by decreasing fold change in the hamster sera. Dots correspond to the mean fold change, lines to the estimated 95% HPDI. Fold change is shown on the log_2_ scale. (*D*) Posterior distributions of the estimated slope explained by whether a titer was measured using hamster (green) or human (red) sera, as well as the contrast of these distributions (Human–Hamster). The dot corresponds to the mean, the bar to the 95% HPDI. (*E*) Antigenic map constructed from a subset of the hamster data. (*F*) Antigenic map constructed using the human sera data. In panels *E* and *F*, the arrows point to the position of the variants in the human and hamster maps, respectively.

To assess whether the experimental hamster infections generated similar responses to human first infections, we compared a subset of hamster titrations to those performed using first-infection human sera. Due to the absence of sera from humans with first infections by Omicron variants, we could only compare sera of individuals infected with D614G, Alpha, and Beta, titrated against the D614G, Alpha, Beta, and Delta variants. We found overall higher titers among the hamster sera (Fig. 3 *A* and *B*). To estimate overall differences in titer magnitude between human and hamster sera, we modeled each titer as a combination of the GMT for a variant and serum group, a reactivity bias of each serum to account for overall higher or lower immune responses per individual, and a magnitude effect estimated separately for hamster and human sera. Fig. 3*B* shows the posterior distribution of the magnitude effect, as well as the difference of the magnitude effect between human and hamster sera. Fold change was similar in terms of rank order of the variants (Fig. 3 *C* and *D*), with the exception of D614G and Delta in Beta sera. To estimate overall differences in the magnitude of fold change between human and hamster sera, the fold change was estimated as a combination of the average fold change from the homologous variant measured for each variant in a serum group multiplied by a human- or hamster-specific slope effect. Fig. 3*D* shows the posterior distribution of the slope effect, as well as the difference of the slope effect between human and hamster sera. The hamster sera overall measure less fold change compared to the human sera. The antigenic maps generated from the hamster and human sera show similar relative positioning of the variants, however, in accordance with the lower fold change measured in the hamster sera, the variants in the hamster antigenic map are positioned closer together (Fig. 3 *E* and *F*).

## Discussion

Here, we present an antigenic map of SARS-CoV-2 variants using sera from hamsters infected twice with viruses selected from a set of eight SARS-CoV-2 variants. The antigenic map shows a cluster of pre-Omicron variants and distinct positions of the Omicron variants. The JN.1 variant is positioned furthest from the D614G variant and away from the previously circulating EG.5.1 and XBB.2 variants. Interestingly, the antigenic map suggests that JN.1 is antigenically similar to the BN.1.3.1 variant, which peaked in circulation globally in December 2022/January 2023. Homologous sera raised against these variants would be required to further assess the antigenic similarity of these variants. Several substitutions have been shown to be primarily responsible of the escape of the JN.1 ancestor BA.2.86 from BA.2, including K356T, N460K, V483del, A484K, F486P, P621S, and ins16MPLF (41, 42). BN.1.3.1 shares two of these substitutions (K356T and N460K), which may explain its apparent antigenic similarity to JN.1.

The antigenic map shown here is similar to that published by Mykytyn et al., which also uses hamster sera (34, 38). Exceptions are the placement of the BA.5 variant closer to BA.2, possibly caused by the absence of sera raised against the BA.2 variant in that study, and the different placements of the XBB.1 and XBB.2 variants in the two maps (these variants differ at positions 252 and 253 of the spike protein). The hamster sera in Mykytyn et al. were raised by infecting hamsters once, with a dose of either 1.0 × 10^5^ or 5.0 × 10^4^ PFU, while the hamsters used here were infected twice with a priming dose of 1.0 × 10^3^ PFU followed by a booster dose of 1.0 × 10^6^ PFU. A prime-boost immunization regimen is likely to alter mature antibody specificity and affinity, resulting in antigenic map differences.

Our comparison of a subset of the hamster sera titrations to titrations done using human sera in the same assay (Fig. 3) shows higher titer magnitude in hamsters and similar rank order of variants when considering fold change, with the exception of the Beta variant in D614G sera, where hamster sera do not replicate the reduction in titers observed in human sera. The magnitude of fold change was larger in the human sera, possibly because the hamsters were infected twice. The antigenic maps, while similar in topology, were more spread out when based on human sera. Two studies have also assessed whether titers measured with hamster sera correspond to those measured with human sera. One compared a set of 18 diverse neutralization datasets generated in different laboratories using different assays (43), while the other performed titrations of hamster and human sera using the same assay in the same laboratory (44). Both found generally higher titers in the hamster compared to the human sera and similar fold changes in terms of magnitude and the rank order of variants. Similar trends with regard to the increased escape of the Beta variant in D614G sera in human sera compared to hamster sera are also seen in these studies. When compared to first-infection human sera, the maps based on hamster sera presented here and in ref. 38 agree in their tighter clustering of the pre-Omicron variants and larger differences between the Omicron subvariants (7–9, 11, 13, 43). The human sera maps show larger relative differences among the pre-Omicron variants and a tighter placement of the Omicron variants. The tighter clustering of the pre-Omicron variants in the hamster sera data may be due to a more standardized immunization regimen in hamsters. Furthermore, the human sera antigenic maps have been generated predominantly employing lentivirus pseudotype or Focus Reduction Neutralization assays, while the hamster maps used PRNTs. As the antigenic distance between the Omicron variants is mainly governed by Omicron sera, the smaller distances observed among the Omicron variants in human sera maps may be caused by the smaller number of Omicron than pre-Omicron sera in those maps, as well as generally lower titers generated by infection with those variants in infection-naive humans.

For robust placement of variants in an antigenic map, it is advantageous if the sera are antigenically diverse. This is highlighted by the finding that the antigenic diversity between the Omicron subvariants is much less visible on maps made exclusively from pre-Omicron sera (*SI Appendix*, Fig. S15 *A*–*C*). Generally, it was possible to reduce the number of serum groups in an antigenic map consisting of the pre-Omicron and early Omicron (BA.1, BA.2, and BA.4/BA.5) variants to as few as three, provided that the three variants against which the sera were raised were antigenically diverse. However, a reduction in serum group size to less than three sera led to increased distortion of the antigenic map (*SI Appendix*, Fig. S20). To reduce the number of experimental animals, an informed selection of immunization strains rather than reduction of animal group size is advisable.

Our study has the following potential limitations. First, the three hamsters infected per variant may not depict the full variability of individual responses to the different variants. We cannot assess here to what extent variability of individual responses in hamsters reflects that of humans. Second, the prime-boost strategy we used for hamster immunizations may have influenced affinity maturation, possibly affecting the relative neutralization titers between variants measured here. Third, while the antigenic map presented here is broadly similar to other maps, there are some differences. For example, the higher than homologous titers of BF.7 and BQ.1.18 as well as the higher titers of XBB.2 compared to BN.1.3.1 in BA.5 sera disagree with data published by Mykytyn et al. (38). Some of the variability may be caused by the use of different live virus isolates in different laboratories, in particular substitutions affecting furin cleavage that are acquired during virus passaging and which may affect plaque size and counts (45). Also, we performed titrations on Vero E6 cells that do not express TMPRSS2, which may lead to slightly different neutralization patterns in variants favoring TMPRSS2-dependent entry (even though neutralization mainly depends on receptor engagement) (46). Variations between datasets highlight the importance of performing antigenic surveillance in multiple laboratories using different assays.

With the majority of the population possessing a variable degree of immunity against SARS-CoV-2, there is a clear and pressing need to develop an animal first-infection sera model system to measure SARS-CoV-2 antigenic variation. The data presented here underscore the suitability and utility of using monospecific hamster sera. Continually extended antigenic maps will be invaluable for maintaining an understanding of SARS-CoV-2 antigenic evolution and will aid vaccine strain selection decisions.

## Materials and Methods

### Cell Cultures and Viruses.

Vero E6 (ATCC CRL-1586) and Calu-3 (HTB-55) cells were maintained at 37 °C, 5% CO_2_ in culture medium Dulbecco’s Modified Eagle’s Medium (DMEM, ThermoFisher Scientific) supplemented with 10% fetal bovine serum (FBS, ThermoFisher Scientific), 1% nonessential amino acids (ThermoFisher Scientific), and 1% sodium pyruvate 100 mM (NaP, ThermoFisher Scientific). All cell lines tested negative for Simian virus 5 (*Orthorubulavirus mammalis*) and mycoplasma contaminations. Low passage virus stocks were generated in Vero E6 or Calu-3 cells and sequenced to investigate the presence of substitutions introduced during isolation and passaging. Results are given in *SI Appendix,* Table S1. The B.1+E484K virus was generated using the transformation-associated recombination (TAR) cloning method to introduce an E484K exchange into an infectious SARS-CoV-2 cDNA clone (47, 48).

### Animal Husbandry.

Six- to ten-wk-old female Syrian hamsters (*Mesocricetus auratus*; breed RjHan:AURA) were purchased from Janvier Labs and kept in groups of 1 to 3 animals in individually ventilated cages (GR900; Tecniplast). Animals were provided with bountiful enrichment (Carfil) and had unrestricted access to food and water at all times. Cage temperatures ranged from 22 to 24 °C and relative humidity ranged from 40 to 55%. All animal work was performed in a certified BSL-3 facility. Hamsters arrived at the facility 7 d prior to the start of the experiments.

### Generation of Hamster Sera.

For each SARS-CoV-2 variant (D614G, Beta, Delta, B.1+E484K, Omicron BA.1, Omicron BA.2 (two isolates), Omicron BA.5, Omicron XBB.2, *SI Appendix,* Table S1), three hamsters were intranasally infected to generate defined immune sera directed against an individual virus variant. Two individuals were infected with the Alpha variant. To minimize infection-induced distress and at the same time maximize serum antibodies, hamsters were infected using a two-stage protocol. Each animal was initially infected with 1,000 plaque forming units (PFU) of the respective variant and allowed to recover from infection for 21 d. On day 21 after initial infection, animals were reinfected with 1 × 10^6^ PFU of the same variant. All infections were carried out intranasally under general anesthesia and in a total volume of 60 μL as previously described (49). Fourteen days after reinfection, animals were humanely killed and blood was collected for serum harvest. During the experiment, all hamsters were monitored twice daily for clinical signs of disease in compliance with a state-authority approved animal use protocol (Landesamt für Gesundheit und Soziales in Berlin, Germany, Approval No. 0086/20).

### Human Sera.

Human sera were available through previous studies (47, 50) on antibody response after mild to moderate COVID-19. Sera were taken before the circulation of any SARS-CoV-2 variants of concern (47) and from Alpha (47) and Beta (50) infected patients. D614G sera were taken approximately 6 wk postinfection, Alpha sera approximately 2 wk postinfection, and Beta sera a mean of 39 d (range: 12 to 78 d) post positive PCR.

### PRNTs.

The neutralizing activity of each serum against the different virus strains was determined by PRNT, largely as described before (51). In short, Vero E6 cells (1.6 × 10^5^ cells/well) were seeded in 24-well plates and incubated for ~24 h. Hamster sera were serially diluted in OptiPro medium and mixed with medium containing 100 PFU of the respective virus, incubated at 37 °C for 1 h and then added to the Vero E6 cells in duplicate. After a further hour at 37 °C, supernatants were discarded, the cells washed once with PBS, and supplemented with 1.2% Avicel solution in DMEM. After 3 d at 37 °C, the supernatants were removed, the plates were fixed using a 6% formaldehyde/PBS solution and stained with crystal violet. All serum dilutions were tested in duplicates. Plaques were counted for each well. The virus strains used were Munich isolate 984, D614G (GISAID ID: EPI_ISL_406862), Alpha (EPI_ISL_754174), Beta (EPI_ISL_862149), Delta (EPI_ISL_3710429), Omicron BA.1 (EPI_ISL_7019047), Omicron BA.2 CSpecVir26729_2 (EPI_ISL_9553926), Omicron BA.2 CSpecVir26729_12 (EPI_ISL_9553935), Omicron BA.4 (EPI_ISL_16221624), Omicron BA.5 (EPI_ISL_16221625), Omicron BF.7 (EPI_ISL_17293602), Omicron BQ.1.18 (EPI_ISL_17293256), Omicron XBB.2 (EPI_ISL_17548230), Omicron BN.1.3.1 (EPI_ISL_17549547), Omicron EG.5.1 (EPI_ISL_18233735), and Omicron JN.1 (EPI_ISL_18474148).

### Analysis of Neutralization Titers.

We investigated different methods for inferring titers from the raw plaque count data. This included assessing the effects of using different plaque reduction criteria/sensitivity levels (NT50, NT75, NT90, or NT99 titers) (*SI Appendix*, Figs. S1–S3), as well as assessing the effect of estimating titers either by rounding to the closest dilution (“discrete titers”), or by fitting a sigmoid neutralization curve (“continuous titers”) (*SI Appendix,* Figs. S4 and S5). Discrete titers were determined as the dilution closest to where X% of all plaques are neutralized, where X corresponds to the sensitivity level (e.g., 50 for NT50 titers). Continuous titers were inferred using the HillCurve function in the neutcurve package, version 0.5.7 (39) with and without constraining the lower and/or upper ends of the neutralization curve to zero or one, respectively.

For the analysis of reactivity patterns of different hamster serum groups and for antigenic cartography, NT90 titers were determined from the raw plaque counts (Dataset S2) using the neutcurve package (version 0.5.7) (39) written in Python, constraining the lower end of the neutralization curve to zero (*SI Appendix*, *Titer Determination*). We used titers determined with the lower and upper end of the neutralization curve constrained at zero and one, respectively, for the 28 pairs of sera and variants listed in *SI Appendix,* Table S2. Final titers are given in *SI Appendix,* Table S3. For the human sera, titers were determined at a sensitivity level of 50, rounded to the closest dilution.

Geometric mean titers (GMT) and fold changes were estimated using the gmt and log2diff functions, respectively, in the titertools package (52) in R (version 4.2.0) (53), as described in ref. 11. The method takes into account nondetectable titers for the calculation of GMTs and fold changes in a Bayesian framework. We used the following parameters: ci_method = “HDI” (highest density interval), ci_level = 0.95, dilution_stepsize = 0, the prior for the mean was a normal distribution with mean of 0 and SD of 100, the prior for the SD was an inverse gamma distribution with a shape parameter of 2 and a scale parameter of 0.75.

For the comparison of hamster and human sera, we estimated titer magnitude and fold change explained by the hamster and human sera, as described in ref. 43. Briefly, titer magnitude was estimated such that the logged titer for variant *i* and serum *j* in serum group *J* and dataset *m* is given by *logtiter_ijm_ = serumGroupGMT_iJ_ + serumEffect_j_ + datasetMagnitudeEffect_m_ + e*, where *serumGroupGMT* corresponds to the GMT calculated across all sera of a serum group *J* titrated against a variant, *serumEffect* corresponds to the reactivity bias of serum *j*, and *datasetMagnitudeEffect* corresponds to the effect of the dataset (hamster, human) on the logtiter. Fold change relative to the homologous variant measured by variant *i* and serum j in serum group J in dataset m was estimated as predictedFoldchange_ijm_ = agFoldchange_iJ_ * datasetSlope_m_ + e, where *agFoldchange* corresponds to the fold change from the homologous variant for variant *i* in serum group *J* and *datasetSlope* corresponds to the additional slope explained by the hamster or the human dataset *m*. *e* is independently and normally distributed log titer noise. Models were implemented in stan and fitted using “cmdstanr” (54). The Markov chain Monte Carlo was run with four chains with 10,000 iterations and 1,000 warmup iterations per chain. Convergence was assessed by ascertaining that the Rhat statistic is below 1.01 and by inspecting the traces.

### Antigenic Cartography.

Briefly, in an antigenic map optimization, the target distance between each variant and serum pair is set to the difference between the log_2_ highest overall titer of the serum and the log_2_ titer of a given variant. Therefore, the variant with the highest titer against a particular serum will have a target distance of 0. The positions of the variants and sera in the antigenic map are adjusted in an optimization process that aims to minimize the sum of the squared differences between the target distances and the corresponding distances in the antigenic map (28). Antigenic maps were inferred using Racmacs version 1.1.39 (40). All antigenic maps were constructed with a dilution_stepsize of 0 for maps made from continuous titers or 1 for maps made from discrete titers. The maps were optimized in two dimensions for 500 optimizations and the minimum column basis parameter set to “none.” We performed several analyses to assess the dimensionality of the antigenic map (*SI Appendix*, Fig. S10), how well the distances in the antigenic map represent the target distances (*SI Appendix,* Figs. S11 and S12), the robustness of the map to titer noise (*SI Appendix,* Fig. S13 *A*–*C*) and missing titers (*SI Appendix*, Figs. S13 *D*–*F* and S14), and the predictive power of the antigenic map (*SI Appendix*, Figs. S17 and S18). These analyses are described in *SI Appendix*, *Assessing Antigenic Map Model Fit*.

## Supplementary Material

Appendix 01 (PDF)

Dataset S01 (CSV)

Dataset S02 (PNG)

## Data Availability

Neutralization data and code used in the analyses presented in this publication can be found on GitHub (55) and Zenodo (56).
