# Supplementary material for: Antigenic cartography using variant-specific hamster sera reveals substantial antigenic variation among Omicron subvariants
Source: Proc Natl Acad Sci U S A. 2024 Jul 30;121(32):e2310917121. doi: 10.1073/pnas.2310917121 (PMC11317614; doi:10.1073/pnas.2310917121)
Supplement: Supplementary file 1 — Appendix 01 (PDF) [file pnas.2310917121.sapp.pdf]

Supporting information for

Antigenic cartography using variant-specific hamster sera reveals substantial antigenic variation among Omicron subvariants

Barbara Mühlemann, Jakob Trimpert, Felix Walper, Marie L. Schmidt, Jenny Jansen, Simon Schroeder, Lara M. Jeworowski, Jörn Beheim-Schwarzbach, Tobias Bleicker, Daniela Niemeyer, Anja Richter, Julia M. Adler, Ricardo Martin Vidal, Christine Langner, Daria Vladimirova, Samuel H. Wilks, Derek J. Smith, Mathias Voß, Lea Paltzow, Christina Martínez Christophersen, Ruben Rose, Andi Krumbholz, Terry C. Jones, Victor M. Corman, Christian Drosten

Christian Drosten, Institute of Virology, Charité – Universitätsmedizin Berlin
Emai: christian.drosten@charite.de

This PDF file includes:

Supporting text
Figures S1 to S20
Tables S1 to S3

Other supporting materials for this manuscript include the following:

Datasets S1 to S2

Supplementary Text

Titer determination

Effects of using discrete and continuous titers

Visual inspection of the neutralization curves in Dataset 11 showed that continuous titers inferred when fixing the neutralization curve at zero provide the most parsimonious fit to the data. Given the overall similarity of the fold change and antigenic maps estimated by the different methods, we therefore proceeded to use continuous titers inferred when fixing the neutralization curve at zero. However, visual inspection of the neutralization curves in Dataset 1 also indicated 28 titers (2.1 vs Alpha, 2.2 vs BA.2_2, 3.2 vs BA.2_12, 3.3 vs Alpha, 3.3 vs Beta, 4.1 vs Alpha, 4.2 vs Delta, 4.3 vs Alpha, 4.3 vs Delta, 5.1 vs Alpha, 5.1 vs BA.2_12, 5.2 vs Alpha, 5.2 vs BA.2_2, 5.3 vs BA.2_2, 6.1 vs BA.2_2, 6.2 vs BA.2_2, 7.1 vs D614G, 7.1 vs Alpha, 7.1 vs Delta, 7.1 vs E484K, 7.1 vs BA.2_12, 7.1 vs Mu, 7.2 vs Mu, 7.3 vs Alpha, 7.3 vs Mu, 8.1 vs BA.2_12, 9.1 vs BF.7, 9.3 vs BQ.1.18) where continuous titers inferred when fixing the lower and upper ends of the neutralization curve at zero and one, respectively, provided a better fit to the data. These titers were therefore adapted as shown in Table S2. Antigenic maps with and without these adapted titers only show minimal differences (SI Appendix fig. S6, RMSD = 0.13). The final titers used for further analyses are shown in Table S3.

Assessing antigenic map model fit

We performed several analyses to assess the dimensionality of the antigenic map (SI Appendix, fig. S10), how well the distances in the antigenic map represent the measured titers (SI Appendix, figs. S11, S12), the robustness of the map to titer noise (SI Appendix, fig. S13A-C) and missing titers (SI Appendix, figs. S14-15), and the predictive power of the antigenic map (SI Appendix, figs. S16-S18).

In order to assess the dimensionality of the antigenic map, we investigated how well the antigenic distances in maps optimized in different dimensions fit the measured titers. To this end, we performed 1000 repeats where 10% of the titers were excluded at random, the map re-optimised using 500 optimisations in 1-5 dimensions, and calculated the root-mean-squared error between the measured titers and the predicted titers in the antigenic map. The map optimized in three dimensions fit the data best (RMSE of detectable titers = 1.08), but only performed marginally better than maps optimized in two (RMSE of detectable titers = 1.13), four (RMSE of detectable titers = 1.10), and five (RMSE of detectable titers = 1.11)

dimensions (SI Appendix, fig. S10C). The overall arrangement of the variants in a map optimized in three dimensions closely resembles the arrangement of variants optimized in two dimensions (SI Appendix, fig. S10A, B). For ease of representation, we show the map in two dimensions.

We next assessed how well the distances fitted in the antigenic map represent the measured distances inferred from the titers (SI Appendix, figs. S11, S12). Overall, there was a good fit between the fitted and the measured titers. The difference between the fitted and measured titers was 0.03 on the $\log_2$ scale, with a standard deviation (sd) of 0.73. When assuming a mean of 0, the standard deviation is 0.79. The standard deviation was higher than the standard deviation observed between repeated titrations (sd = 0.47) (SI Appendix, fig. S9). However, because the repeats were not independent, the repeat variation may be underestimated, and the difference between fitted and measured titers is comparable to that observed in other studies investigating SARS-CoV-2 antigenic variation (*1*, *2*). We also found no evidence of increased differences between fitted and measured titers for specific serum groups and variant pairs (SI Appendix, fig. S12).

We next investigated the robustness of the map to measurement error, variant reactivity biases and missing data. To assess robustness to measurement error and variant reactivity biases we performed a bootstrap analysis where normally distributed noise was added to the titer and / or variant reactivity (SI Appendix, fig. S13A-C). We performed 1000 bootstrap repeats of adding random noise and summarized the positional variation of the variants and sera, such that the areas shown in SI Appendix, fig. S13 represent 68% (one standard deviation) of the positional variation of each variant or serum. Random noise added to the titers had a standard deviation of 0.33, as estimated from the standard deviation of differences between repeat titrations (SI Appendix, fig. S9). Random noise added to variant reactivity had a standard deviation of 0.4, following Wilks et al., 2022 (*1*). In general, positions were largely robust to the addition of random noise, in particular for the Omicron BA.1, BA.2, BA.4/BA.5, BF.7, BQ.1.18 and XBB.2 variants, and larger positional uncertainty within the pre-Omicron variants and for Omicron BN.1.3.1, EG.5.1, and JN.1. Next, we assessed the robustness of the antigenic map to the removal of each variant (SI Appendix, fig. S14A) and serum group (SI Appendix, fig. S14B) in turn. Variant positions were largely robust to the removal of individual variants, with increased variation observed for the Omicron BA.1, Omicron BA.5 and the joint removal of both Omicron BA.2 variants (SI Appendix, fig. S14A). Similarly, variant positions were stable to the removal of serum groups, albeit with slightly larger variation when removing the BA.1 and BA.5 sera (SI Appendix, fig. S14B).

Finally, we investigated how well the map is able to predict missing titrations. To this end, we performed 500 cross-validation repeats, where 10% of the titers were removed at random, the map re-optimised, and the missing titers predicted from the antigenic map. The mean difference between the predicted and measured titer was 0.52, with a standard deviation of 1.43 when assuming a mean of 0. This is higher than the difference between fitted and measured titers (SI Appendix, fig. S11, mean = 0.03, standard deviation = 0.79 when assuming a mean of 0 and only considering detectable titers). We visualized the difference between predicted and measured titers split by variant and serum group, and found that no variant is predicted to consistently have higher residuals in all serum groups.

Supplementary figures

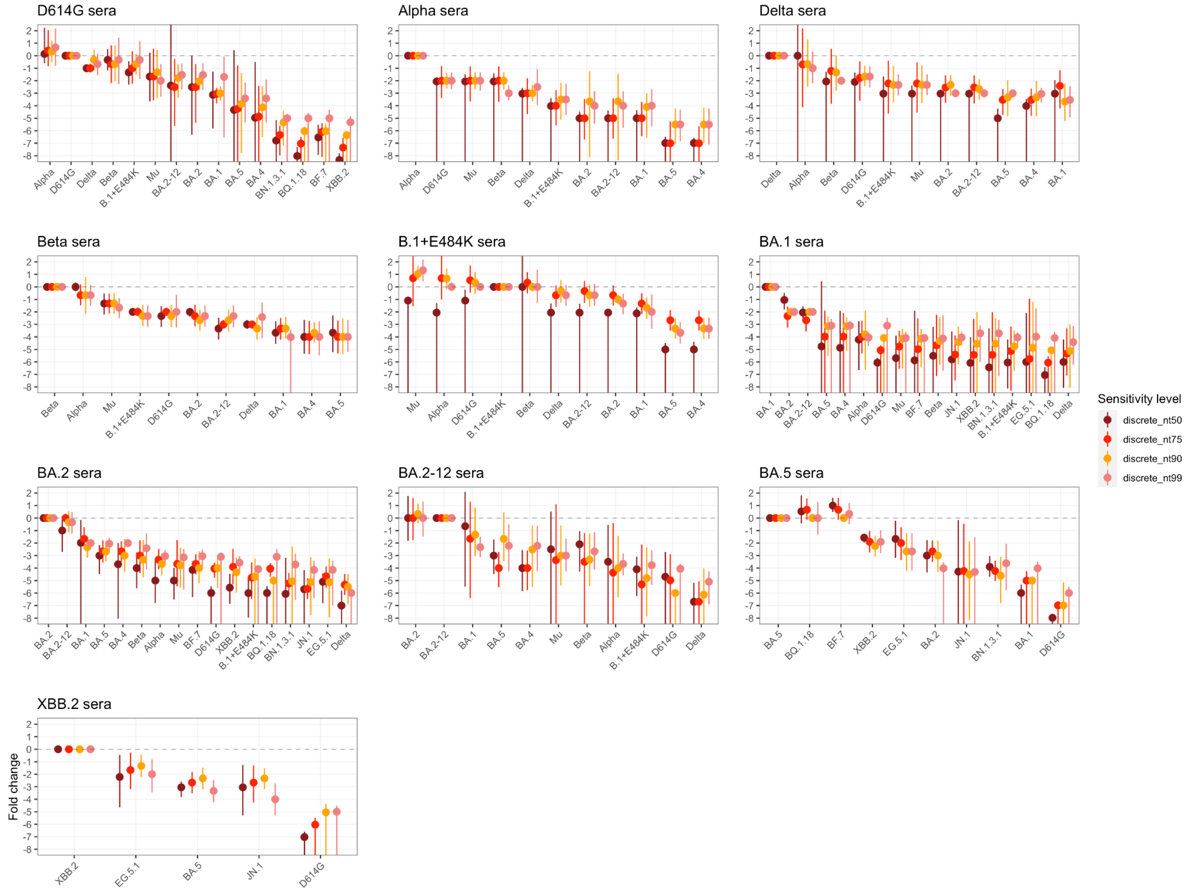

Figure S1: Fold change measured when inferring discrete titers with different sensitivity levels. Larger fold changes with wide confidence intervals result when titers are non-detectable. Fold changes are largely comparable between sensitivity levels. Dots show the estimated mean fold change, with lines showing the 95% highest posterior density interval. Ratio of titers have been log-transformed.

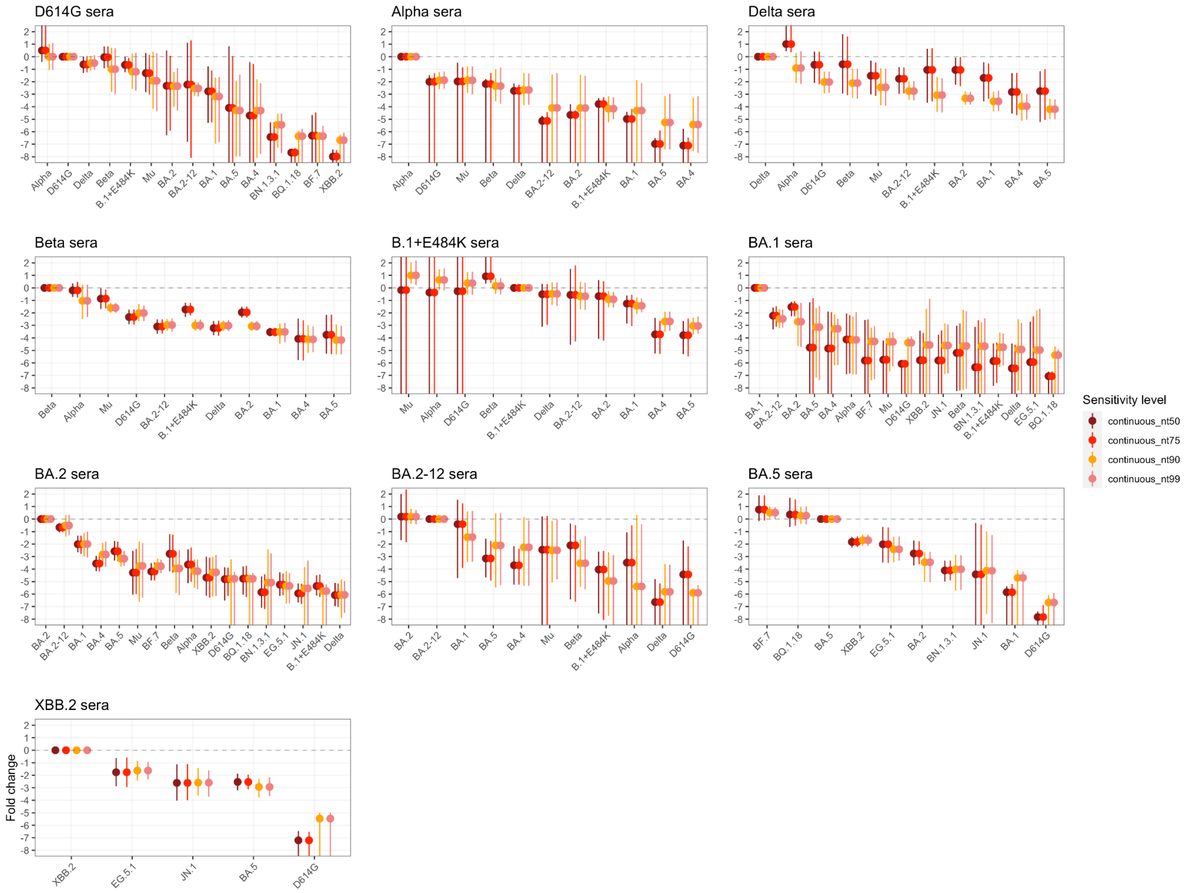

Figure S2: Fold change measured when inferring continuous titers with different sensitivity levels. Larger fold changes with wide confidence intervals result when titers are non-detectable. Fold changes are largely comparable between sensitivity levels. Continuous titers were inferred by constraining the neutralization curve at zero and one. Dots show the estimated mean fold change, with lines showing the 95% highest posterior density interval. Ratio of titers have been log-transformed.

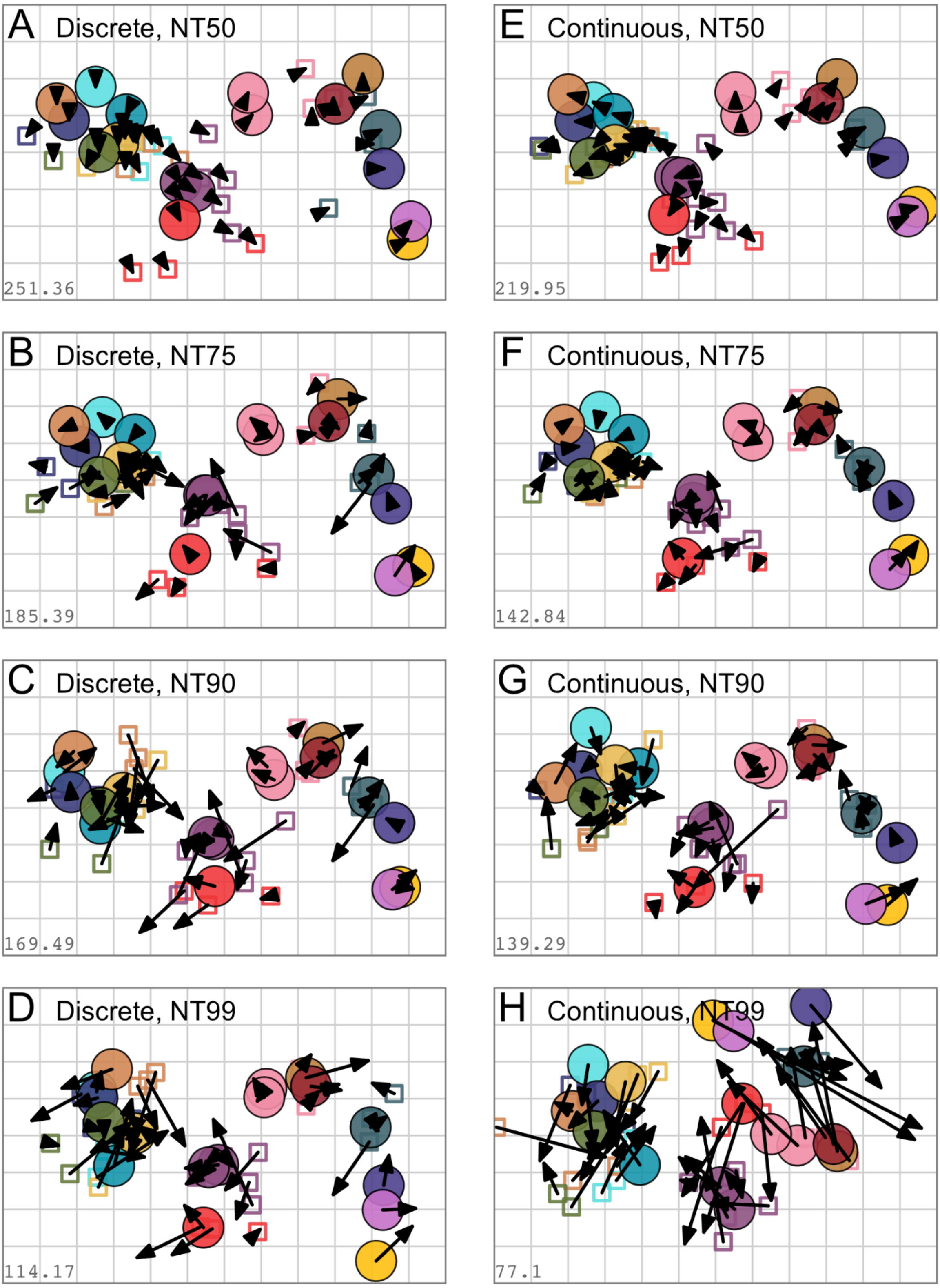

A Discrete, NT50
251.36
B Discrete, NT75
185.39
C Discrete, NT90
169.49
D Discrete, NT99
114.17
E Continuous, NT50
219.95
F Continuous, NT75
142.84
G Continuous, NT90
139.29
H Continuous, NT99
77.1

Figure S3: Antigenic maps made from discrete (left) and continuous (right) titers inferred with different sensitivity levels. Antigenic maps were optimized in two dimensions, with 500 optimisation and the minimum column basis parameter set to 'none'. A-D) Maps made with discrete titers using a dilution_stepsize of 1. E-H) Maps made with continuous titers using a dilution_stepsize of 0. Arrows point to the position of the same variant and serum in the NT50 map shown in panel A for panels A-D, and panels E for panels E-H. A,E) NT50, B,F) NT75, C,G) NT90, D,H) NT99. Viruses are shown as circles, sera as squares, with sera colored by the color of their homologous variant (blue: D614G, green: Alpha, dark-yellow: Beta, orange: Delta, green-blue: Mu, cyan: B.1+E484K, red: BA.1, orchid: BA.2 (2x, on top of each other), pink: BA.4 and BA.5, ochre: BQ.1.18, maroon: BF.7, sea-green: XBB.2, light-orchid: BN.1.3.1, dark blue: EG.5.1, yellow: JN.1). The gray number at the bottom of the plot indicates the sum of the squared difference between the distance of each variant and serum in the titer table and the antigenic map.

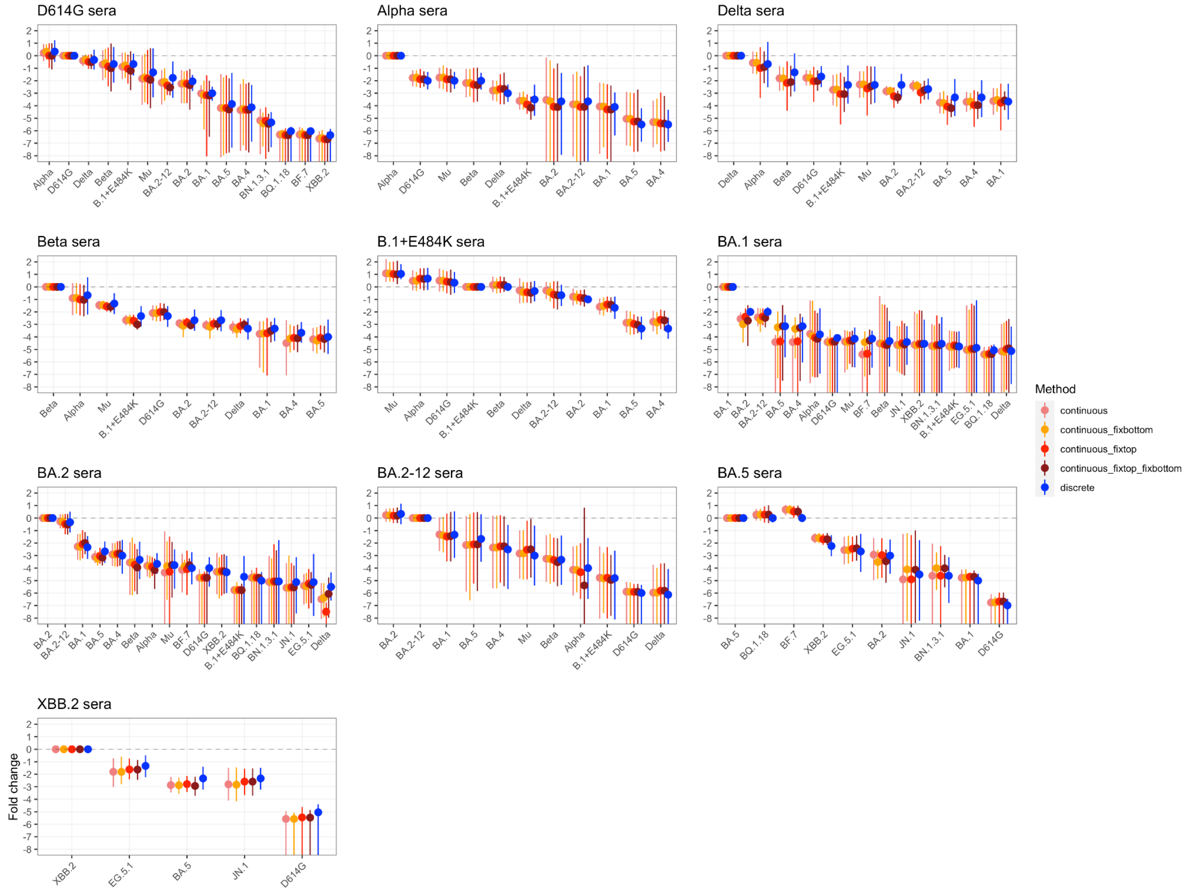

Figure S4: Fold change measured when inferring titers using either continuous or discrete methods at NT90. Continuous titers were inferred fixing the bottom and top of the neutralization curve at zero and one, respectively (continuous_fixtop_fixbottom), only fixing the top of the neutralization curve (continuous_fixtop), only fixing the bottom of the neutralization curve (continuous_fixbottom), not fixing the neutralization curve (continuous), and using discrete titers (discrete). Fold changes are similar between different methods. Dots show the estimated fold change, with lines showing the 95% highest posterior density interval. Ratio of titers have been log-transformed.

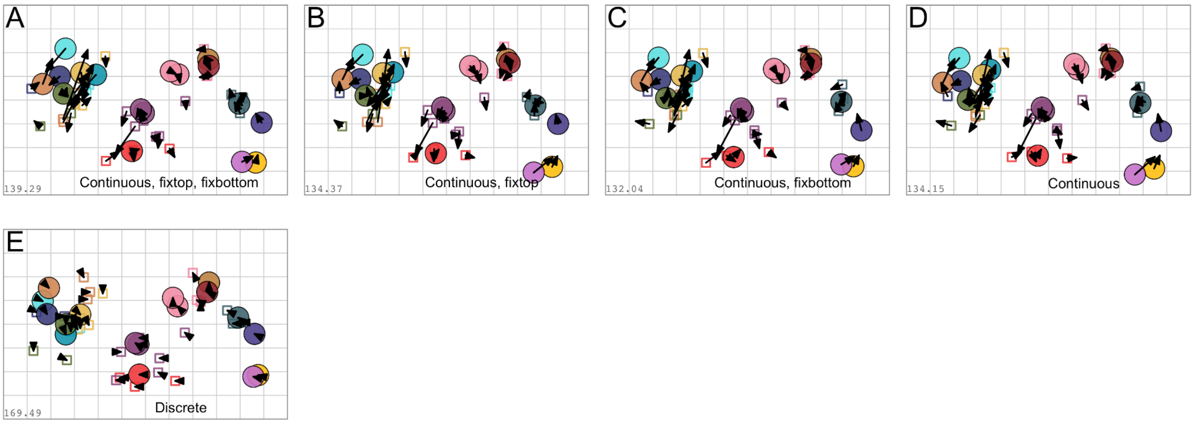

Figure S5: Comparison of discrete and continuous NT90 maps. In each map, the arrows point to the position of the variant and serum in the antigenic map made from discrete titers, shown in panel E. A) Map made from continuous titers, fixing the bottom and top of the neutralization curve at zero and 1, respectively. B) Map made from continuous titers, fixing the top of the neutralization curve at one. C) Map made from continuous titers, fixing the bottom of the neutralization curve at one. D) Map made from continuous titers, without fixing the neutralization curve. E) Map made from discrete titers. Viruses are shown as circles, sera as squares, with sera colored by the color of their homologous variant (blue: D614G, green: Alpha, dark-yellow: Beta, orange: Delta, green-blue: Mu, cyan: B.1+E484K, red: BA.1, orchid: BA.2 (2x, on top of each other), pink: BA.4 and BA.5, ochre: BQ.1.18, maroon: BF.7, sea-green: XBB.2, light-orchid: BN.1.3.1, dark blue: EG.5.1, yellow: JN.1). The gray number at the bottom of the plot indicates the sum of the squared difference between the distance of each variant and serum in the titer table and the antigenic map.

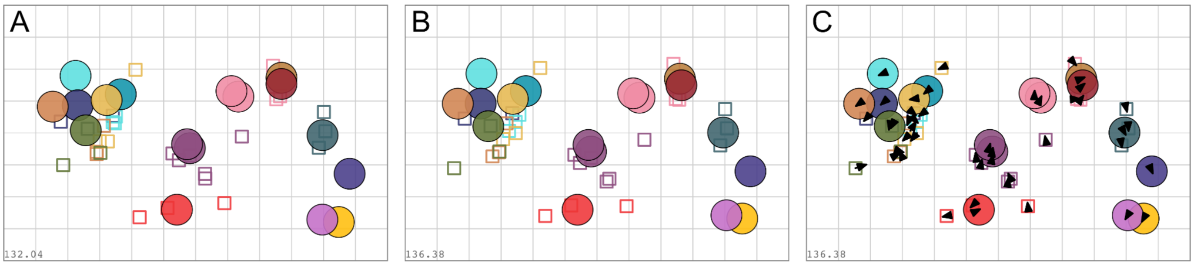

Figure S6: Comparison of the PRNT90 fixbottom map and the PRNT90 fixbottom map with a subset of titers manually adapted. A) PRNT90 map inferred from titers calculated while fixing the bottom of the neutralization curve at zero (‘fixbottom map’). B) PRNT90 map inferred from titers calculated while fixing the bottom of the neutralization curve at zero, with a subset of titers manually adapted. C) PRNT90 fixbottom map with a subset of titers manually adapted with arrows pointing to the positions of sera and variants in the non-adapted map. The adapted titers were: 2.1 vs Alpha, 2.2 vs BA.2_2, 3.2 vs BA.2_12, 3.3 vs Alpha, 3.3 vs Beta, 4.1 vs Alpha, 4.2 vs Delta, 4.3 vs Alpha, 4.3 vs Delta, 5.1 vs Alpha, 5.1 vs BA.2_12, 5.2 vs Alpha, 5.2 vs BA.2_2, 5.3 vs BA.2_2, 6.1 vs BA.2_2, 6.2 vs BA.2_2, 7.1 vs D614G, 7.1 vs Alpha, 7.1 vs Delta, 7.1 vs E484K, 7.1 vs BA.2_12, 7.1 vs Mu, 7.2 vs Mu, 7.3 vs Alpha, 7.3 vs Mu, 8.1 vs BA.2_12, 9.1 vs BF.7, 9.3 vs BQ.1.18. Adaptations were made as shown in Table S2. Viruses are shown as circles, sera as squares, with sera colored by the color of their homologous variant (blue: D614G, green: Alpha, dark-yellow: Beta, orange: Delta, green-blue: Mu, cyan: B.1+E484K, red: BA.1, orchid: BA.2 (2x, on top of each other), pink: BA.4 and BA.5, ochre: BQ.1.18, maroon: BF.7, sea-green: XBB.2, light-orchid: BN.1.3.1, dark blue: EG.5.1, yellow: JN.1). The gray number at the bottom of the plot indicates the sum of the squared difference between the distance of each variant and serum in the titer table and the antigenic map.

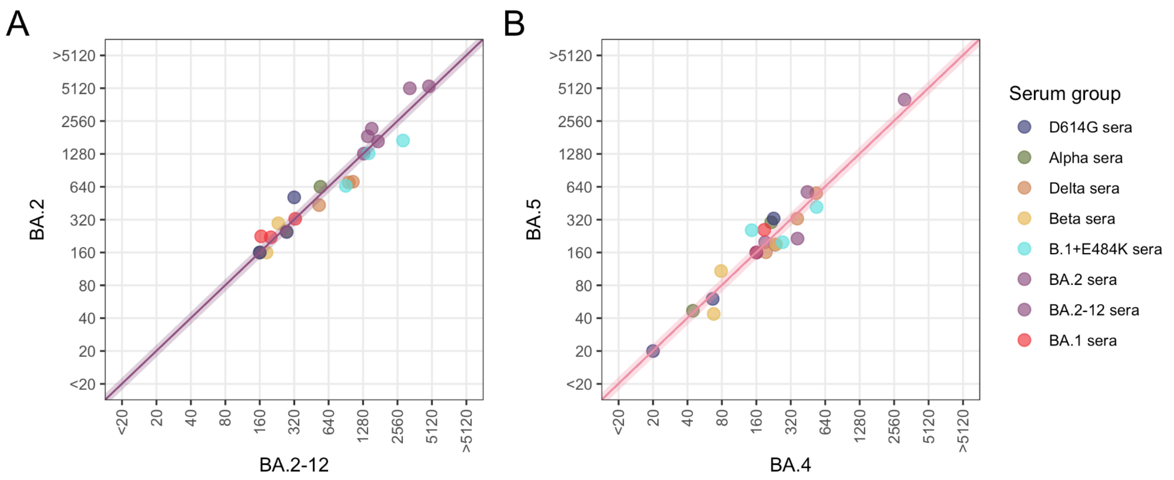

Figure S7: Pairwise comparison of titers of two variants expected to be antigenically similar. A) Comparison of the titers from the two different BA.2 isolates. The mean fold difference between the two variants is 1 (95% highest posterior density interval: -1.14, 1.13). B) Comparison of the titers from the BA.4 and BA.5 variants. The mean fold difference between the two variants is -1 (95% highest posterior density interval: -1.16, 1.15). Dots are colored by serum group. The intercept of the colored line shows the mean fold difference between the titers from the two variants, the slope is equal to 1. The shaded area shows the 95% posterior density interval of the fold difference.

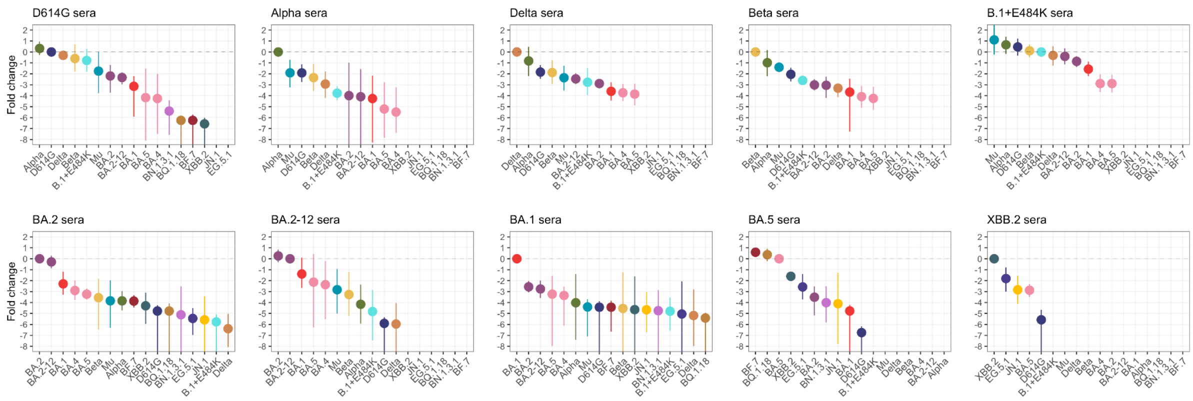

Figure S8: Fold change compared to the homologous variant. Circles show the point estimate for the mean fold change, while the intervals show the 95% highest posterior density interval of the fold change estimate. Variants are ordered by decreasing fold change within each serum group. Ratio of titers have been log-transformed.

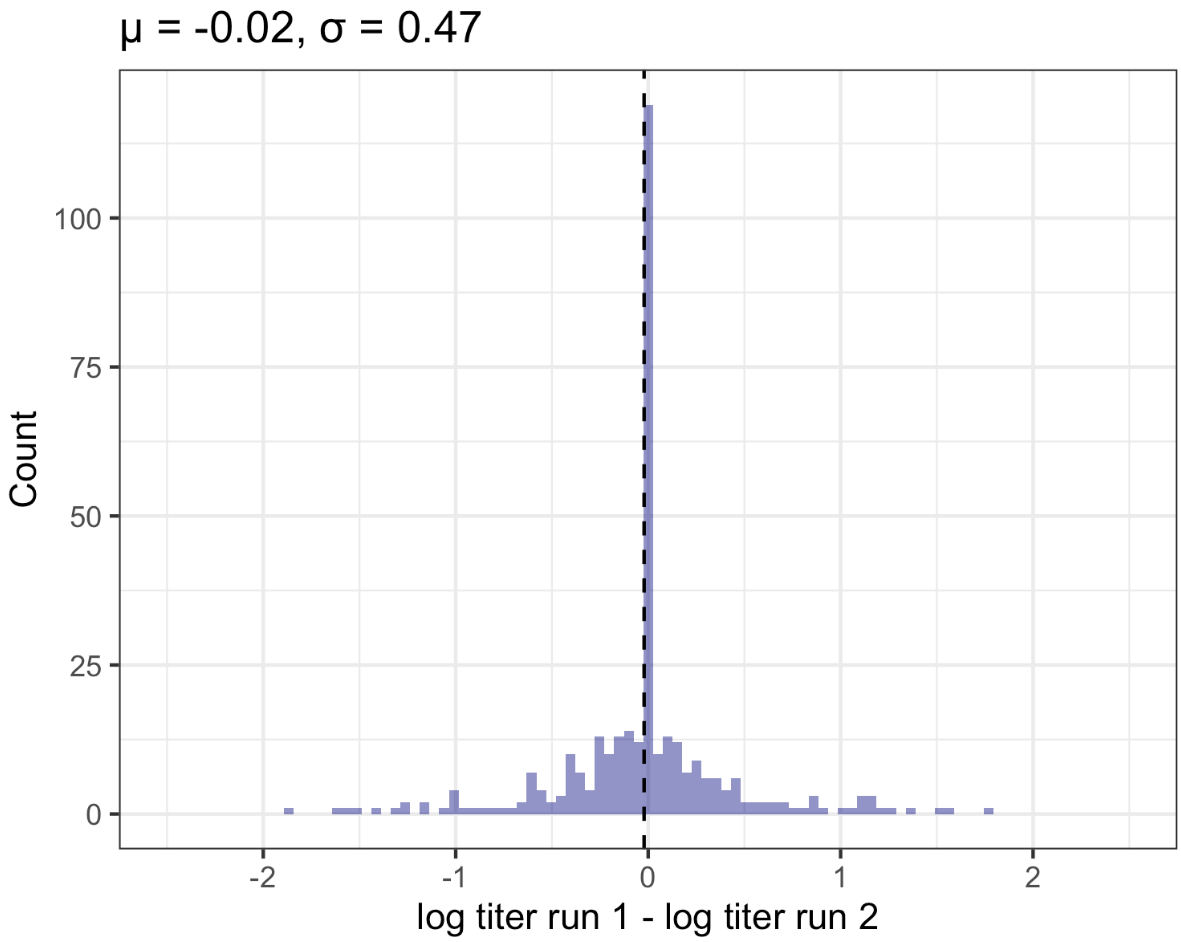

Figure S9: Repeat variation. Each titer was measured in duplicate wells. The figure shows the difference in $\log_2$ titer between the first and the second repeat. Non-detectable titers were set to limit of detection - 1 on the $\log_2$ scale. The mean difference between repeats was 0.02, with a standard deviation of 0.47. Assuming a mean of 0 to account for the systematic bias of titrations between repeats, the standard deviation is 0.47 on the $\log_2$ scale. Accounting for the presence of measurement error in both the first and second titration the standard deviation of noise per measurement is $\sqrt{0.47^2/2} = 0.33$.

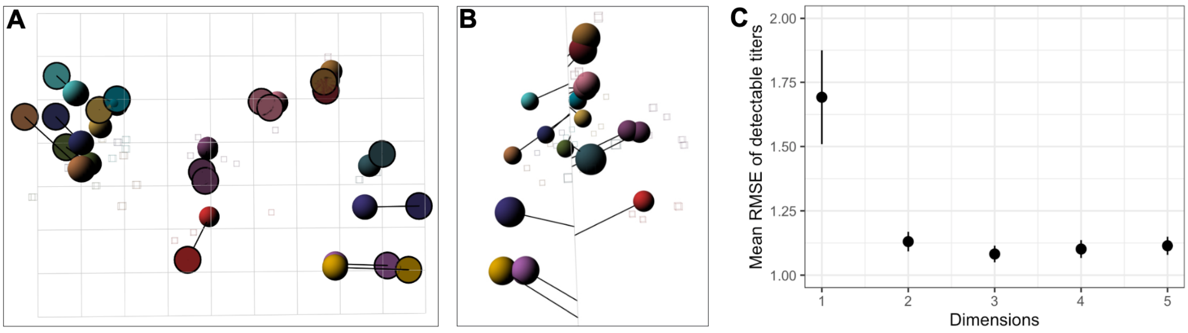

Figure S10: Testing map dimensionality. A and B) The map optimized in three dimensions, shown from the top (A) and from the side (B). The black lines point to the position of the variants in the two-dimensional map shown in Figure 2. Viruses are shown as spheres, sera as cubes, with sera colored by the color of their homologous variant (blue: D614G, green: Alpha, dark-yellow: Beta, orange: Delta, green-blue: Mu, cyan: B.1+E484K, red: BA.1, orchid: BA.2 (2x, on top of each other), pink: BA.4 and BA.5, ochre: BQ.1.18, maroon: BF.7, sea-green: XBB.2, light-orchid: BN.1.3.1, dark blue: EG.5.1, yellow: JN.1). C) Dimensionality test, where 1000 repeats were performed with 10% of titers excluded at random and the map optimized in one to five dimensions. The plot shows the root mean squared error and standard error of the excluded titers between the titers estimated from the map and the known titers.

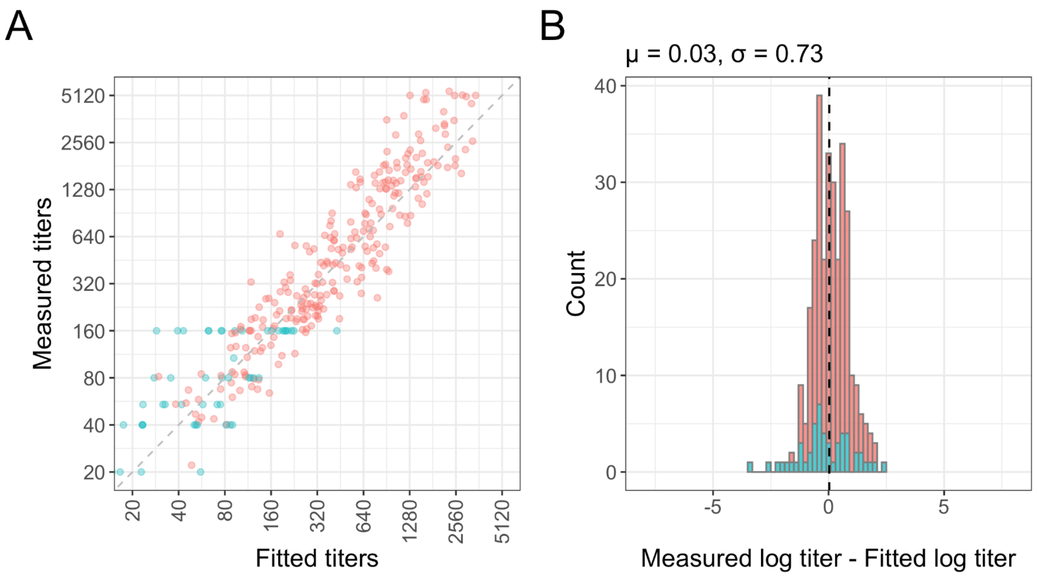

Figure S11: Comparison of measured log_2 titers and fitted log_2 titers as inferred from the antigenic map. Detectable measured titers are shown in red, non-detectable measured titers in blue. A) Scatter plot of measured and fitted log_2 titers. Fitted log_2 titers were inferred from the distances in the antigenic map. B) Histogram of the difference between the measured and the fitted log_2 titers. When measured titers were non-detectable, the residuals were inferred as described in (*1*). The histogram of detectable titers is shown in red, non-detectable titers in blue. Variant and serum group pairs where all titers were non-detectable were excluded (BA.2 sera vs D614G and B.1+E484K, BA.1 sera vs D614G, BA.2-12 sera vs D614G). Measured titers are on average 0.03 higher than the fitted titers (with a standard deviation of 0.73), with the mean indicated by the dashed black line. Assuming a mean of 0 to account for the systematic bias of titrations between repeats, the standard deviation is 0.79 on the log_2 scale. This is higher than the standard deviation of the variation between repeats, however the standard deviation between repeats may be lower, since the repeats were not independent.

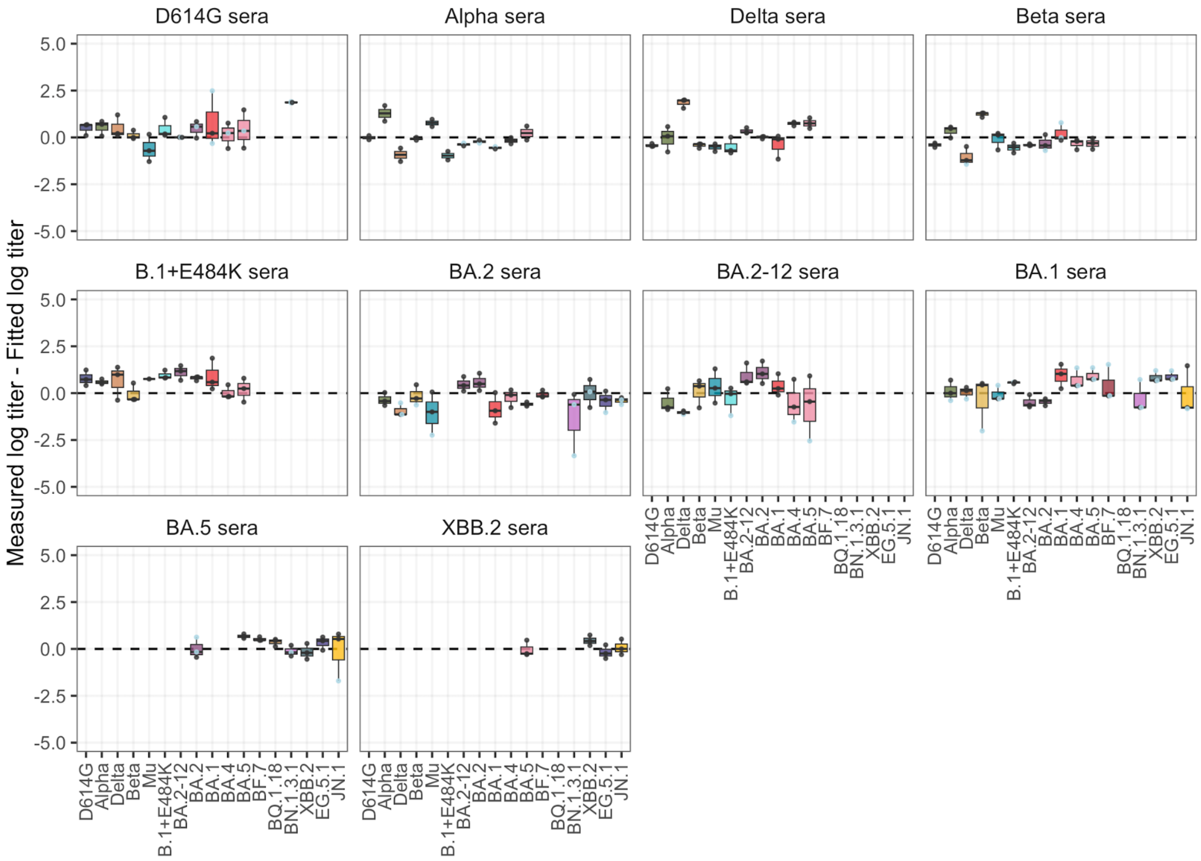

Figure S12: The difference between the measured log_2 titer and the fitted log_2 titer as inferred from the antigenic map, split by serum group and variant. Residuals for detectable titers are shown in black, residuals for non-detectable titers in blue. Variant and serum group pairs where all titers were non-detectable were excluded (BA.2 sera vs D614G and B.1+E484K, BA.1 sera vs D614G, BA.2-12 sera vs D614G). The boxplot indicates the median and 25th and 75th percentile.

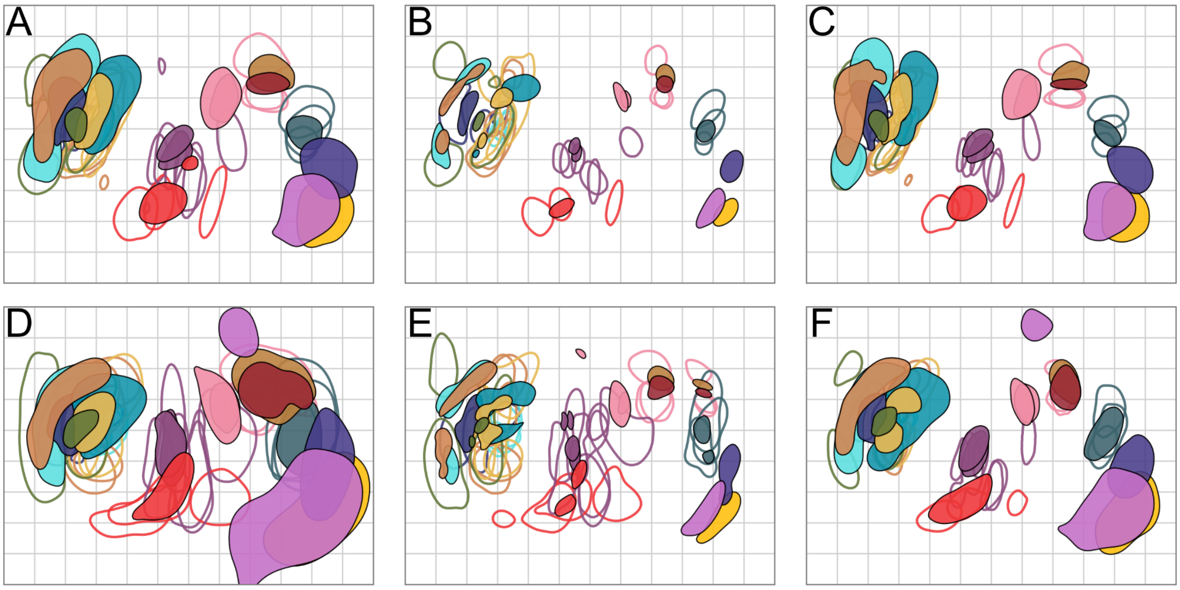

Figure S13: Assessing sensitivity of the antigenic map to measurement error (A-C) and missing titers (D-F). A-C) Sensitivity of antigenic map to measurement error. 1000 bootstrap repeats were performed where normally distributed noise was added to each titer and / or to the titers of each variant. A) Noise added to titers and variant reactivity. B) Noise added to titers only. C) Noise added to variants only. Normally distributed noise added to the titers had a standard deviation of 0.33, following the standard deviation of the measurement error estimated in SI Appendix, fig. S9. Normally distributed noise added on a per-variant basis had a standard deviation of 0.4. D-F) Robustness of the antigenic map to missing titers. 1000 bootstrap repeats were performed, where a random subset of titers was sampled with replacement, and the map re-optimised. A) Sera and variants were re-sampled. B) Variants were resampled. C) Sera were resampled. The colored regions indicate the area where 68% (1 standard deviation) of the positional variation of each variant (filled blobs) and serum (empty blobs) is captured. Circles are colored by variant (blue: D614G, green: Alpha, dark-yellow: Beta, orange: Delta, green-blue: Mu, cyan: B.1+E484K, red: BA.1, orchid: BA.2 (2x, on top of each other), pink: BA.4 and BA.5, ochre: BQ.1.18, maroon: BF.7, sea-green: XBB.2, light-orchid: BN.1.3.1, dark blue: EG.5.1, yellow: JN.1).

A

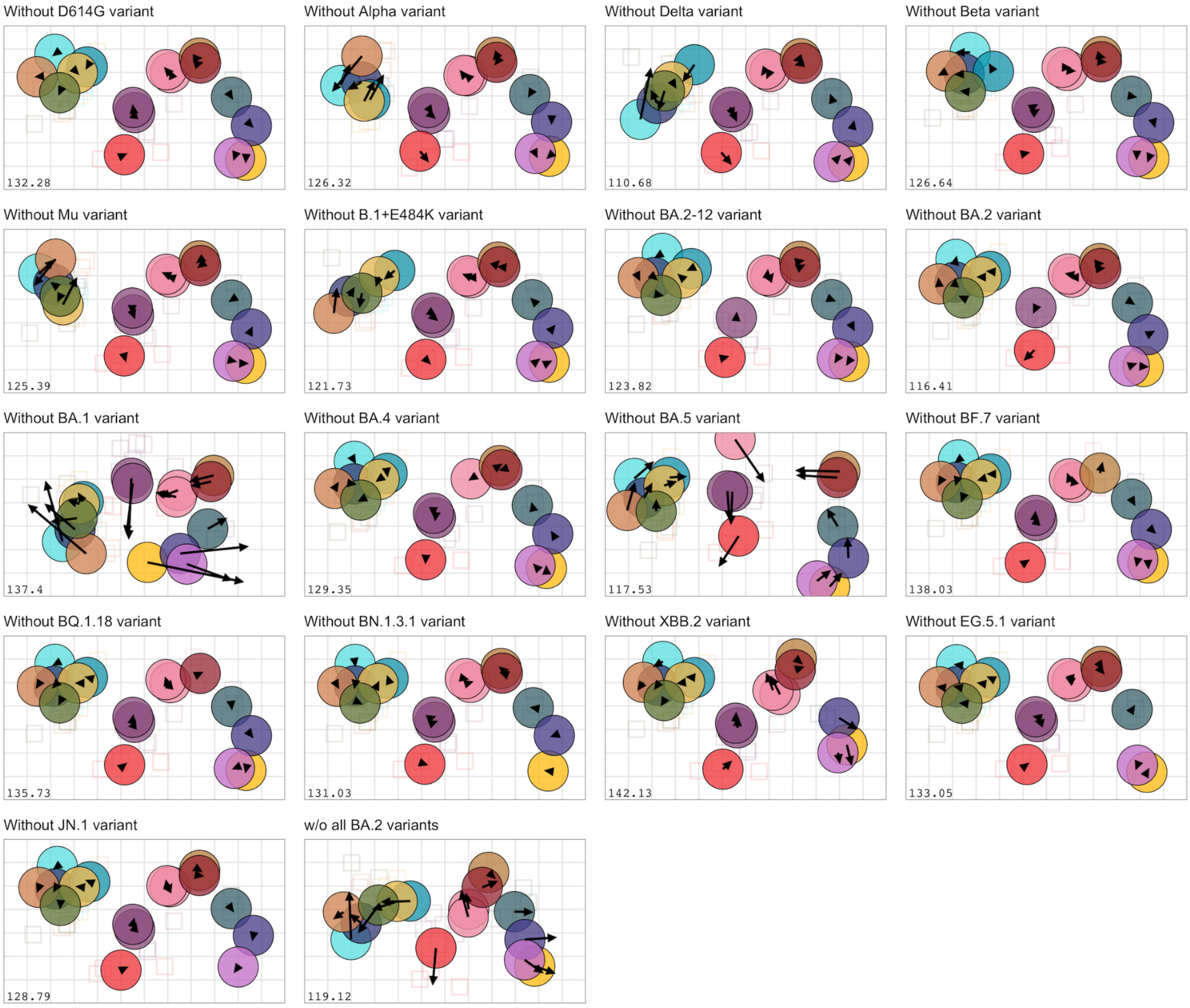
Without D614G variant
132.28
Without Alpha variant
126.32
Without Delta variant
110.68
Without Beta variant
126.64
Without Mu variant
125.39
Without B.1+E484K variant
121.73
Without BA.2-12 variant
123.82
Without BA.2 variant
116.41
Without BA.1 variant
137.4
Without BA.4 variant
129.35
Without BA.5 variant
117.53
Without BF.7 variant
138.03
Without BQ.1.18 variant
135.73
Without BN.1.3.1 variant
131.03
Without XBB.2 variant
142.13
Without EG.5.1 variant
133.05
Without JN.1 variant
128.79
w/o all BA.2 variants
119.12

B

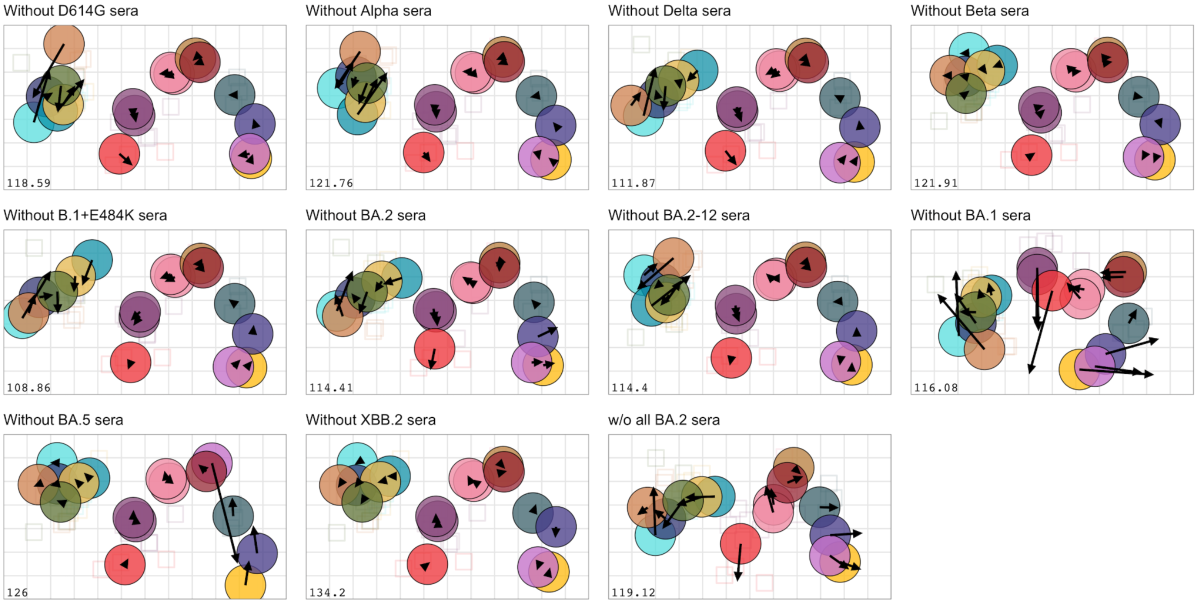

Figure S14: Robustness of the antigenic map to removing variants (A) and serum groups (B). Each variant and serum group was removed in turn, as indicated in the panel title, and the map re-optimised. The arrows point to the position of the variants in the full map, shown in Figure 2. Viruses are shown as circles, sera as squares, with sera colored by the color of their homologous variant (blue: D614G, green: Alpha, dark-yellow: Beta, orange: Delta, green-blue: Mu, cyan: B.1+E484K, red: BA.1, orchid: BA.2 (2x, on top of each other), pink: BA.4 and BA.5, ochre: BQ.1.18, maroon: BF.7, sea-green: XBB.2, light-orchid: BN.1.3.1, dark blue: EG.5.1, yellow: JN.1). The gray number at the bottom of the plot indicates the sum of the squared difference between the distance of each variant and serum in the titer table and the antigenic map.

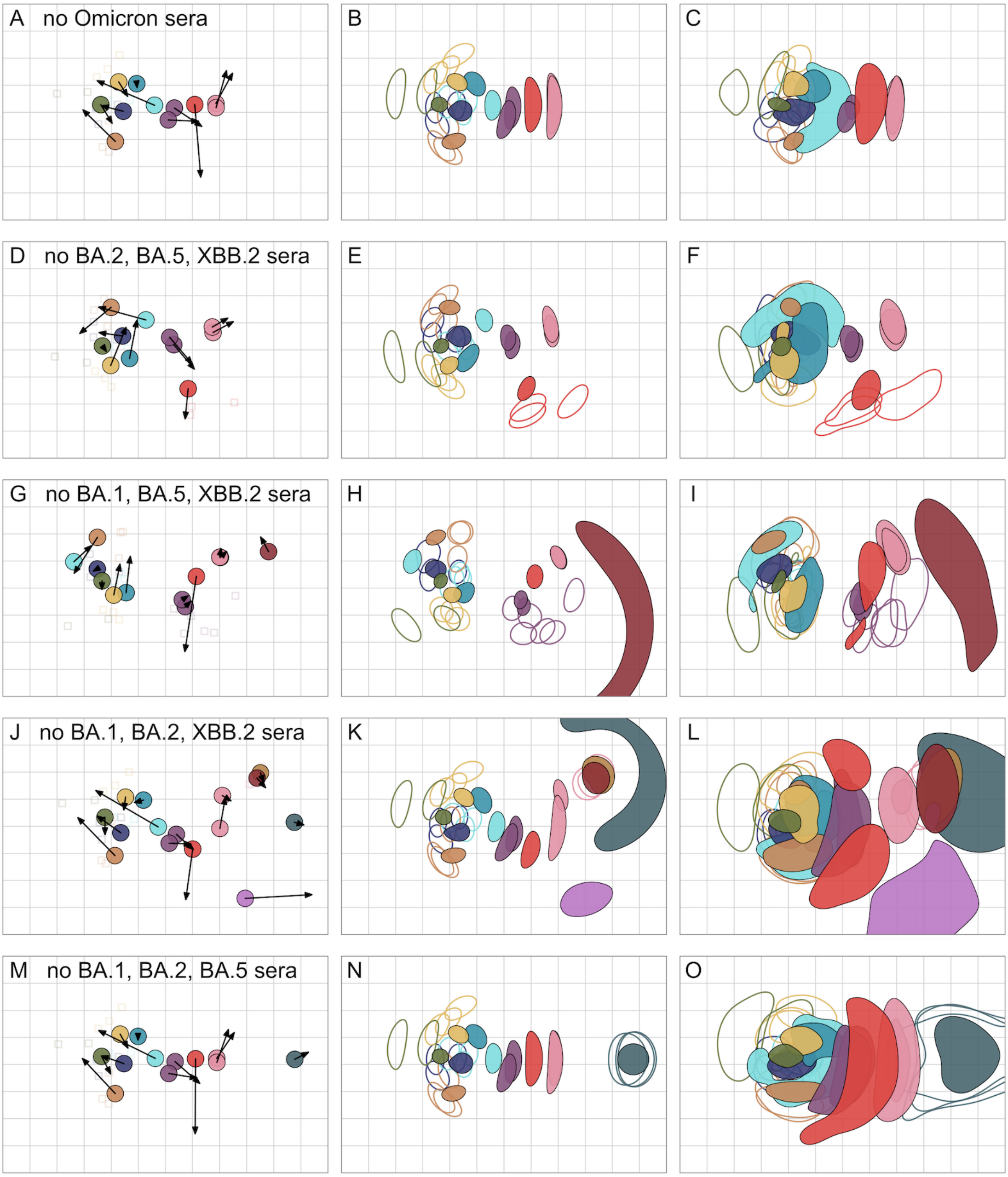

Figure S15: Antigenic map made with different combinations of Omicron sera. The left column always shows the antigenic map inferred without a subset of Omicron sera, with the arrows pointing to the positions of the variants in the full map (as in Figure 2). The middle column shows the antigenic map inferred without a subset of Omicron sera with triangulation blobs. The blobs show the area that each variant (filled blob) or serum (empty blob) could take up without increasing the stress of the map by more than 1. The right column shows the antigenic map inferred without a subset of Omicron sera with resampling bootstrap blobs. An antigenic map was inferred 500 times, and each time titers were sampled with replacement.

The area of the blobs indicate the area that each variant (filled blob) or serum (empty blob) occupy in 68% of the repeats. The coloring of the variants and sera is as in Figure 2 (blue: D614G, green: Alpha, dark-yellow: Beta, orange: Delta, green-blue: Mu, cyan: B.1+E484K, red: BA.1, orchid: BA.2 (2x, on top of each other), pink: BA.4 and BA.5, ochre: BQ.1.18, maroon: BF.7, sea-green: XBB.2, light-orchid: BN.1.3.1, dark blue: EG.5.1, yellow: JN.1). A-C) Antigenic maps without any Omicron sera (the BF.7, BQ.1.18, XBB.2, BN.1.3.1, EG.5.1, and JN.1 variants cannot be placed on the map due to insufficient detectable titers). D-F) Antigenic maps without BA.2, BA.5, and XBB.2 sera (the BF.7, BQ.1.18, XBB.2, BN.1.3.1, EG.5.1, and JN.1 variants cannot be placed on the map due to insufficient detectable titers). G-I) Antigenic maps without BA.1, BA.5, and XBB.2 sera (the BQ.1.1, XBB.2, BN.1.3.1, EG.5.1, and JN.1 variants cannot be placed on the map due to insufficient detectable titers). J-L) Antigenic maps without BA.1, BA.2, and XBB.2 sera (the EG.5.1 and JN.1 variants cannot be placed on the map due to insufficient detectable titers). M-O) Antigenic maps without BA.1, BA.2, and BA.5 sera (the BF.7, BQ.1.18, EG.5.1 and JN.1 variants cannot be placed on the map due to insufficient detectable titers).

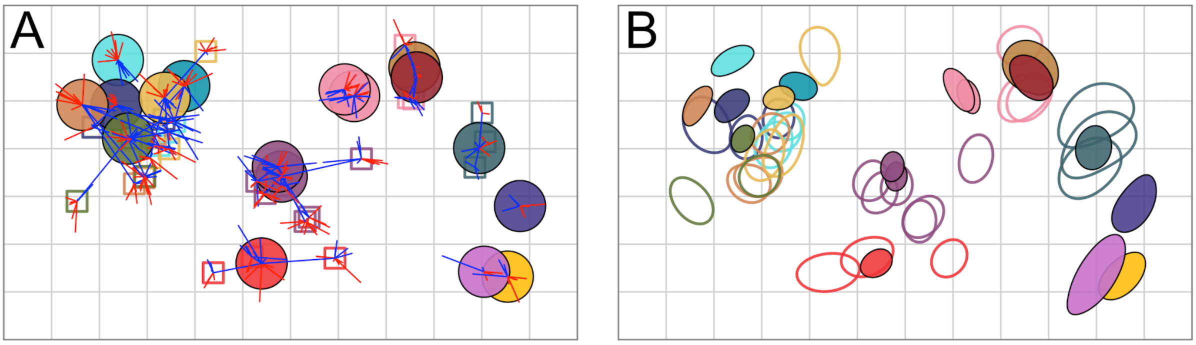

Figure S16: Uncertainty of the position of variants and sera. A) Antigenic map with error lines. For each variant/serum pair a blue or red line is plotted showing the target distance between the variant and serum. A blue line indicates a target distance smaller, a red line a distance larger than the one shown in the map. B) Antigenic map with triangulation blobs. The blobs show the area that each variant (filled blob) or serum (empty blob) could take up without increasing the stress of the map by 1. Circles are colored by variant (blue: D614G, green: Alpha, dark-yellow: Beta, orange: Delta, green-blue: Mu, cyan: B.1+E484K, red: BA.1, orchid: BA.2 (2x, on top of each other), pink: BA.4 and BA.5, ochre: BQ.1.18, maroon: BF.7, sea-green: XBB.2, light-orchid: BN.1.3.1, dark blue: EG.5.1, yellow: JN.1).

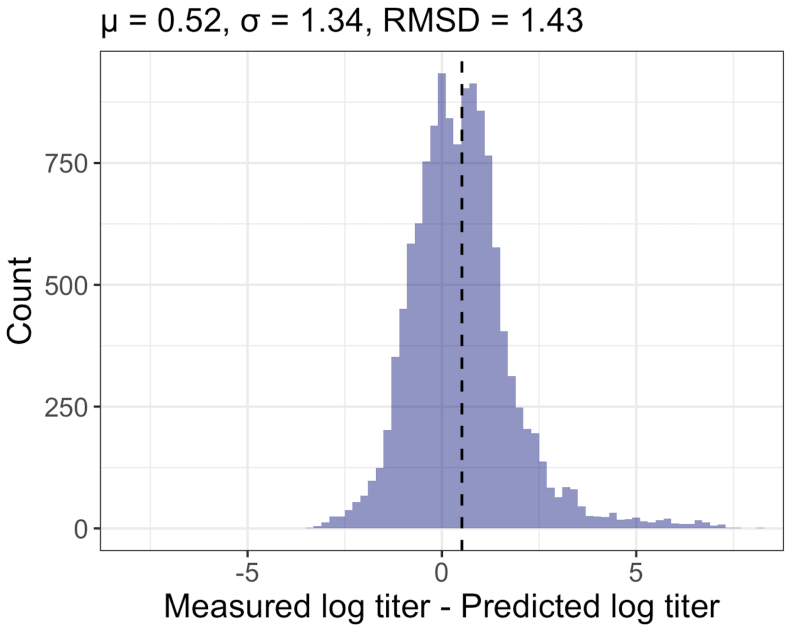

Figure S17: Assessing predictive power of the antigenic map. 500 repeats were performed where the map was re-optimized leaving out 10% of the data at random. The histogram shows the difference between the measured $\log_2$ titer and the predicted $\log_2$ titer as inferred from the map with 10% of the data removed. Only detectable titers were considered. The mean difference between measured and predicted detectable titers on the $\log_2$ scale was 0.52, with a standard deviation of 1.34. Assuming a mean of 0, the standard deviation is 1.43.

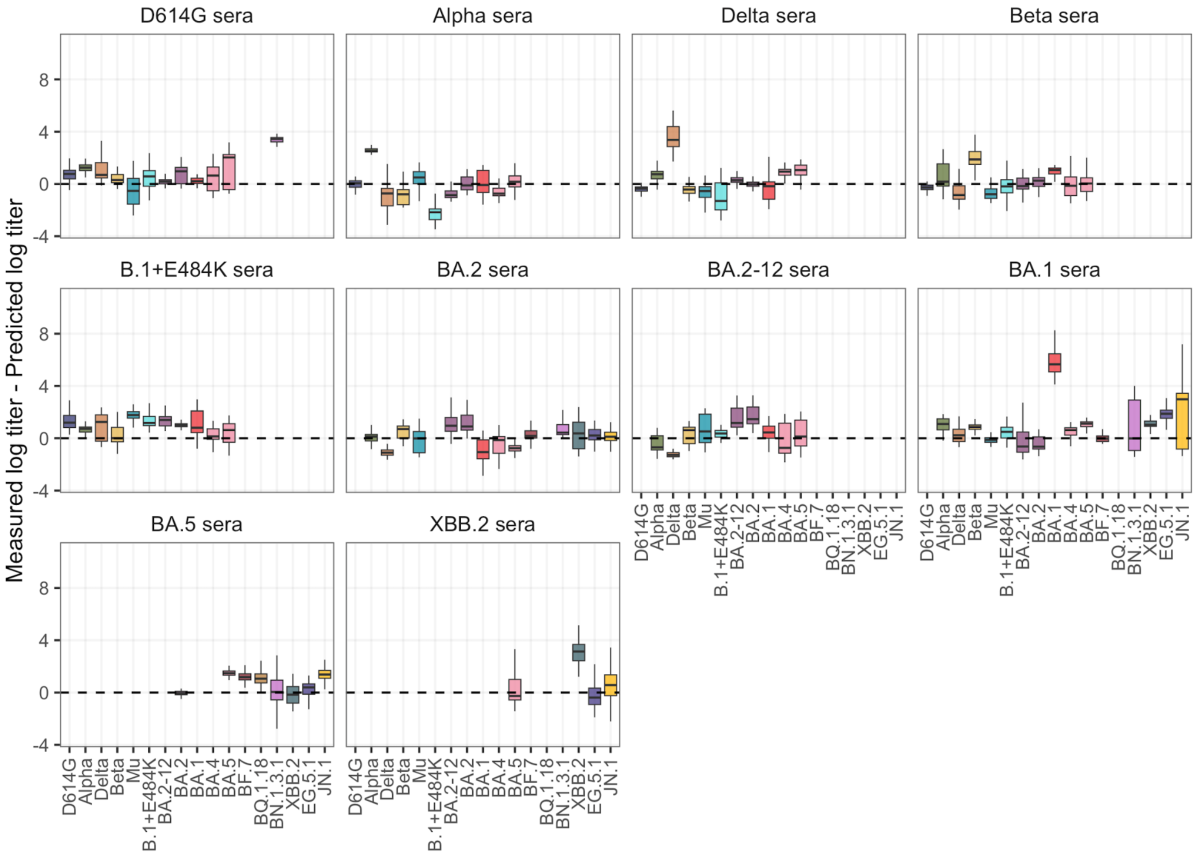

Figure S18: Assessing predictive power of the antigenic map, split by serum group and variant. The difference between measured and predicted log_2 titers was inferred as described in the legend of SI Appendix, fig. S14. Only detectable titers are considered. The boxplot shows the median and 25th and 75th percentile.

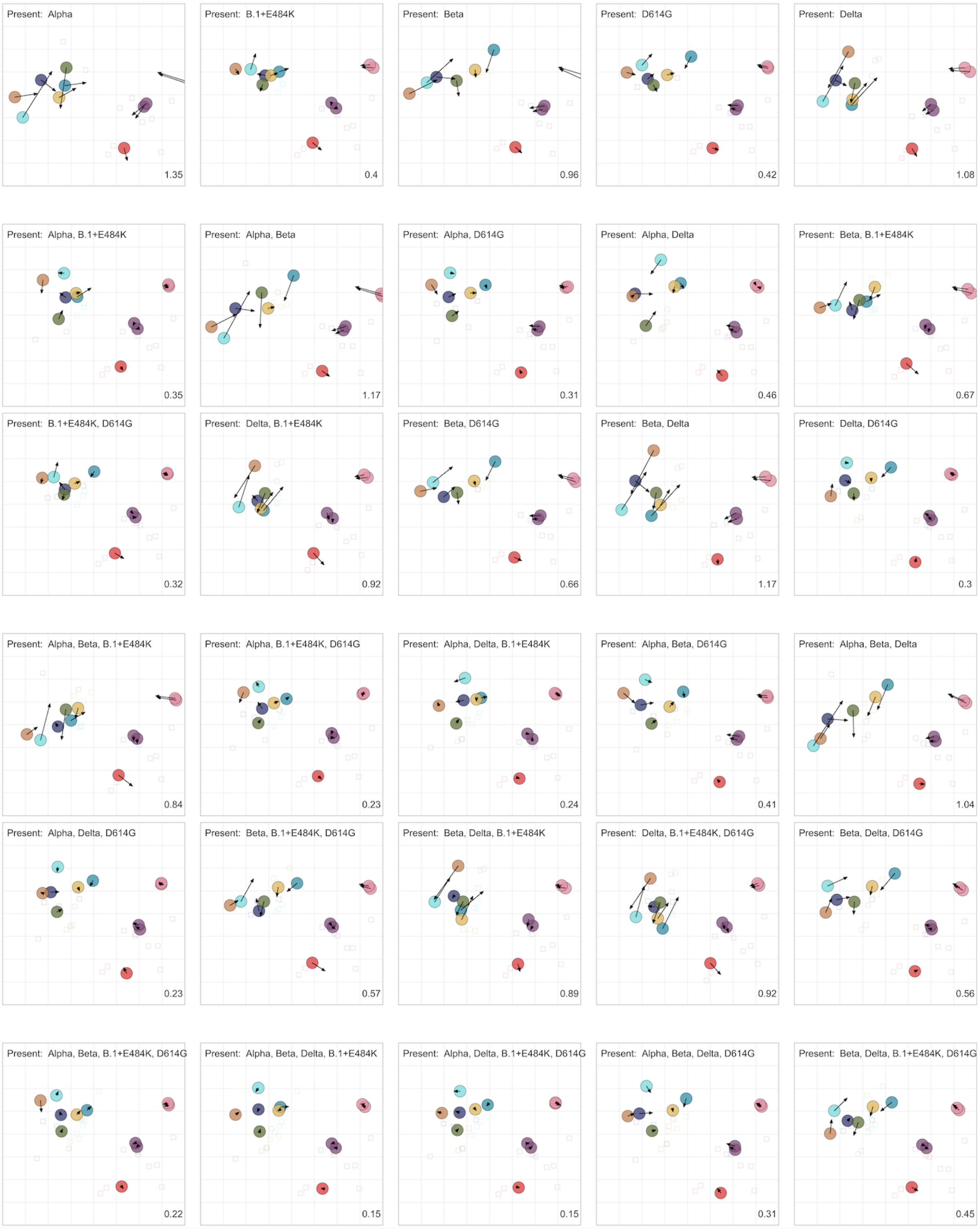
Present: Alpha
1.35
Present: B.1+E484K
0.4
Present: Beta
0.96
Present: D614G
0.42
Present: Delta
1.08
Present: Alpha, B.1+E484K
0.35
Present: Alpha, Beta
1.17
Present: Alpha, D614G
0.31
Present: Alpha, Delta
0.46
Present: Beta, B.1+E484K
0.67
Present: B.1+E484K, D614G
0.32
Present: Delta, B.1+E484K
0.92
Present: Beta, D614G
0.66
Present: Beta, Delta
1.17
Present: Delta, D614G
0.3
Present: Alpha, Beta, B.1+E484K
0.84
Present: Alpha, B.1+E484K, D614G
0.23
Present: Alpha, Delta, B.1+E484K
0.24
Present: Alpha, Beta, D614G
0.41
Present: Alpha, Beta, Delta
1.04
Present: Alpha, Delta, D614G
0.23
Present: Beta, B.1+E484K, D614G
0.57
Present: Beta, Delta, B.1+E484K
0.89
Present: Delta, B.1+E484K, D614G
0.92
Present: Beta, Delta, D614G
0.56
Present: Alpha, Beta, B.1+E484K, D614G
0.22
Present: Alpha, Beta, Delta, B.1+E484K
0.15
Present: Alpha, Delta, B.1+E484K, D614G
0.15
Present: Alpha, Beta, Delta, D614G
0.31
Present: Beta, Delta, B.1+E484K, D614G
0.45

Figure S19: Subsampling pre-Omicron serum groups. Each map was made from the BA.1 and BA.2 sera, and one (first row), two (second and third row), three (fourth and fifth row), or four (bottom row) pre-Omicron serum groups. The arrows point to the position of the variants in a map made from the all-against-all titrated sera and variants (without the Omicron BF.7, BQ.1.18, XBB.2, BN.1.3.1, EG.5.1 and JN.1 variants, and the BA.5 and XBB.2 sera). The number in the bottom right corner shows the total root mean squared deviation of the positions of the variants between the full map and the subsampled map. The text in the top-left indicates the included pre-Omicron serum groups, in addition to the BA.1 and BA.2 serum groups. Each map was made with 100 optimisations. Viruses are shown as circles, sera as squares, with sera colored by the color of their homologous variant (blue: D614G, green: Alpha, dark-yellow: Beta, orange: Delta, green-blue: Mu, cyan: B.1+E484K, red: BA.1, orchid: BA.2 (2x, on top of each other), pink: BA.4 and BA.5). The black number at the bottom right of the plot indicates the sum of the squared difference between the distance of each variant and serum in the titer table and the antigenic map.

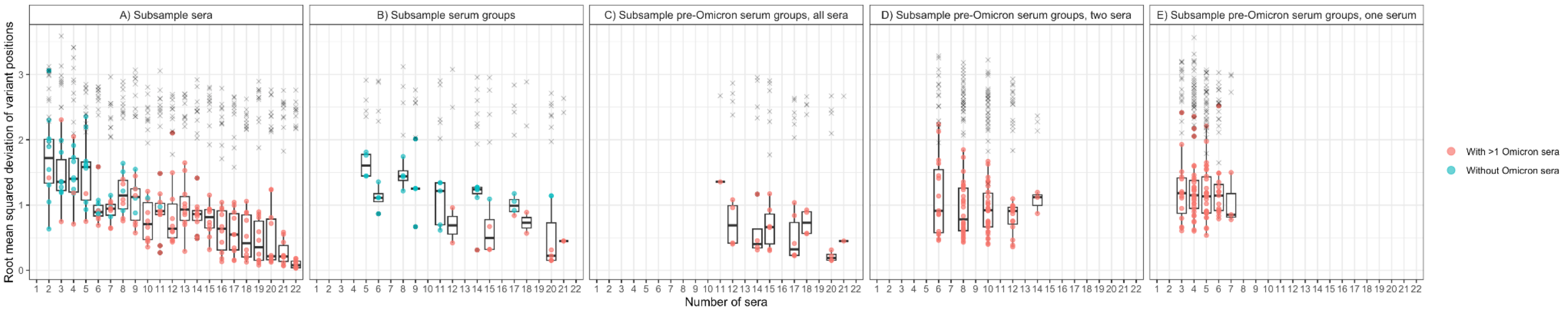

Figure S20: Root mean squared deviation (RMSD) of variant positions of subsampled maps compared to complete map. In order to investigate map stability when reducing the number of sera used, we subsampled a map made from the all-against-all titrated sera and variants (without the Omicron BF.7, BQ.1.18, XBB.2, BN.1.3.1, EG.5.1 and JN.1 variants, and the BA.5 and XBB.2 sera) in different ways: A) Randomly subsample from all sera. B) Randomly sample which serum groups are included. C) Randomly sample which serum groups are included, but always include the BA.1 and BA.2 serum groups. D) Randomly subsample the data in panel C to include two sera per serum group. E) Randomly subsample the data in panel C to include one serum per serum group. A map was constructed from each subsample and the RMSD of the variant positions in the subsampled map compared to a map made from the all-against-all titrated sera and variants was calculated. The RMSD is indicated by boxplots and colored dots, according to the number of sera in each subsampled map. Red dots indicate maps that include both the BA.1 and BA.2 serum groups, blue dots indicate maps that include either the BA.1 or BA.2 or neither of the BA.1 or BA.2 serum groups. To estimate the RMSD under a random scenario, titers in each subsampled map were randomized, a new map constructed and the RMSD from the randomized, subsampled map compared to the full map in Figure 2 was calculated, and is indicated by ‘x’. Subsampled maps were constructed in two dimensions, using 100 optimisations. As the Alpha serum group only includes two sera, and the BA.2 serum groups contain six sera in total, the number of sera in panels B and C is not always a multiple of three.

Supplementary tables

Variant	Spike substitutions relative to Wuhan-Hu-1 (EPI_ISL_402125)	Stock-ID	Additional substitutions and minor variants >5% of reads in S/>20% of reads in rest of the genome	GISAID accession number
D614G	D614G	SARS-CoV-2 984 p2 vs 12.10.2020; FW (V66)	Synonymous minor variant at nt position 11758 (C>T, 5% of reads), 24616 (C>T, 8% of reads), Non-synonymous minor variant at nt position 23525 (S:H655Y, 6% of reads), 23606 (S:R682W, 6% of reads).	EPI_ISL_406862 (original sample sequence), EPI_ISL_16737908 (PRNT stock sequence)
Alpha	H69-, V70-, Y144-, N501Y, A570D, D614G, P681H, T716I, S982A, D1118H	SARS-CoV-2/21528/BaWü p.2 VS 26.03.2021 3dpi, V86	Non-synonymous substitution at nt position 25511 (ORF3a:S40L), 27533 (ORF7a:H47P), 27731 (ORF7a:C113Y) Synonymous substitution at nt position 6379.	EPI_ISL_754174 (original sample sequence), EPI_ISL_16738010 (PRNT stock sequence)
Beta	L18F, D80A, D215G, L241-A243del, K417N, E484K, N501Y, D614G, A701V	SARS-CoV-2/22131/B.1.351 Südafrika p.2, VS 05.02.2021, 3 dpi; AR, V78	Non-synonymous minor variant at nt position 10533 (3CL:C160F, 9% of reads), 10809 (3CL:P252L 19% of reads), 11454 (ORF1a:A3730S, 5% of reads), 11750 (ORF1a:L3829Y, 24% of reads). Non-synonymous substitution at nt position 23607 (S:R682Q), 25563 (ORF3a:Q57H), 25784 (ORF3a:W131L), 27409 (ORF7a:F6I), Synonymous substitution at nt position 6349, Synonymous minor variant at nt position 5392 (28% of reads)	EPI_ISL_862149 (original sample sequence), EPI_ISL_16738012 (PRNT stock sequence)
Delta	T19R, G142D, E156G, F157-, R158-, L452R, T478K, D614G, P681R, D950N	V7_25893_23_C.V.p1	Non-synonymous minor variant at nt position 23606 (S:R682W, 19% of reads).*	EPI_ISL_3710429 (original sample sequence of V7_25893_23_C.V.p1), EPI_ISL_16465138 (PRNT stock sequence)
Omicron BA.1	L5F, A67V, H69-, V70-, T95I, G142D, V143-Y145del, N211I, L212-, -215E, -216P, -217E, G339D, S371L, S373P, S375F, K417N, N440K, G446S, S477N, T478K, E484A, Q493R, G496S, Q498R, N501Y, Y505H, T547K,	V1_26335_C.V.p2	Non-synonymous change in ORF1ab L3829F. Synonymous minor variants at nt positions 17590 and 24382.	EPI_ISL_7019047 (original sample sequence) EPI_ISL_16359096 (PRNT stock sequence)

	D614G, H655Y, N679K, P681H, N764K, D796Y, N856K, Q954H, N969K, L981F			
Omicron BA.2	T19I, L24S, P25-A27del, G142D, V213G, G339D, S371F, S373P, S375F, T376A, D405N, R408S, K417N, N440K, S477N, T478K, E484A, Q493R, Q498R, N501Y, Y505H, D614G, H655Y, N679K, P681H, N764K, D796Y, Q954H, N969K	V2_26729_12_V.V.p6	Non-synonymous change in C3L protease P96H. Synonymous minor variant at nt position 11750. Non-synonymous minor variants at 13131 (ORF1ab:Q4289R), 23606 (S:R682W, 16% of reads), 23757 (S:T732I, 7% of reads), 25618 (ORF3a:G76S, 40% of reads), 26461 (E:L73F, 24% of reads)	EPI_ISL_9553935 (original sample sequence) EPI_ISL_16359856 (PRNT stock sequence)
Omicron BA.2	T19I, L24S, P25-A27del, G142D, V213G, G339D, S371F, S373P, S375F, T376A, D405N, R408S, K417N, N440K, S477N, T478K, E484A, Q493R, Q498R, N501Y, Y505H, D614G, H655Y, N679K, P681H, N764K, D796Y, Q954H, N969K	V3_26729_2_V.V.p6	Non-synonymous change in C3L protease P96H, in E L73F. Non-synonymous minor variant at nt position 2106 (ORF1ab:T614I, 23% of reads), 11355 (ORF1a:A3697V, 23% of reads), 11454 (ORF1a:A3730V 34% of reads), 12789 (ORF1a:T4175I, 29% of reads), 23606 (S:R682W, 8% of reads), 23607 (S:R682L, 16% of reads), 23757 (S:T732I, 34% of reads), 26542 (M:T7I, 28% of reads).	EPI_ISL_9553926 (original sample sequence) EPI_ISL_16359857 (PRNT stock sequence)
rSARS-CoV-2_B.1+E484K	E484K, D614G	B.1 + E484K (p1,VS 07.10.2021) from Simon, V217	Synonymous substitution at nt position 18822. Non-synonymous minor variant at nt position 7493 (ORF1a:M2410L, 26% of reads), 11403 (ORF1a:N3713S), 11522 (ORF1a:F3753V, 7% of reads), 23607 (S:R682P, 19% of reads), synonymous minor variant at nt position 11332 (7% of reads), 29662 (16% of reads)	EPI_ISL_16738016 (PRNT stock sequence)
Omicron BA.4	V3G, T19I, L24S, P25-A27del, H69-, V70-, G142D, V213G, G339D, S371F, S373P, S375F, T376A, D405N, R408S, K417N, N440K, L452R, S477N, T478K, E484A, F486V, Q498R, N501Y, Y505H, D614G, H655Y, N679K,	V38_29061_V.V.p4	Non-synonymous change in nsp6 A161V	EPI_ISL_16221624 (PRNT stock sequence)

	P681H, N764K, D796Y, Q954H, N969K			
Omicron BA.5	T19I, L24S, P25-A27del, H69-, V70-, G142D, V213G, G339D, S371F, S373P, S375F, T376A, D405N, R408S, K417N, N440K, L452R, S477N, T478K, E484A, F486V, Q498R, N501Y, Y505H, D614G, H655Y, N679K, P681H, N764K, D796Y, Q954H, N969K	V34_29057_V.V.p4	Non-synonymous change in membrane glycoprotein V221L	EPI_ISL_16221625 (PRNT stock sequence)
Mu	T95I, Y144T, Y145S, -146N, R346K, E484K, N501Y, D614G, P681H, D950N	V44_29349_V.V.p2	Non-synonymous minor variant at nt positions 23583-23597 (18% of reads with a deletion from aa position S:Y674- to N679-), nt positions 26284-26286 (43% of reads with a deletion of aa position E:V14). Non-synonymous change in ORF3a Q57H, L106F.	EPI_ISL_17566125 (PRNT stock sequence)
Omicron BF.7	T19I, L24S, P25-A27del, H69-, V70-, G142D, V213G, G339D, S371F, S373P, S375F, T376A, D405N, R408S, K417N, N440K, L452R, S477N, T478K, E484A, F486V, Q498R, N501Y, Y505H, D614G, H655Y, N679K, P681H, N764K, D796Y, Q954H, N969K	V66_29620_C.V.p1	Non-synonymous minor variant (28289, N:P6T, 7% of reads), synonymous substitution (28330, A>G), non-synonymous substitution (29253, N:S327L)	EPI_ISL_17293602 (PRNT stock sequence)
Omicron BQ.1.18	T19I, L24S, P25-A27del, H69-, V70-, G142D, V213G, G339D, R346T, S371F, S373P, S375F, T376A, D405N, R408S, K417N, N440K, K444T, L452R, N460K, S477N, T478K, E484A, F486V, Q498R, N501Y, Y505H, D614G, H655Y, N679K, P681H, N764K, D796Y, Q954H, N969K	V60_40617_u.V.p3	Synonymous substitutions in ORF1ab (10156 and 18060), ORF3a (25584), synonymous minor variants (S:24814, T>C (55%)), non-synonymous minor variant in ORF6 (D>L, 51% of reads), non-synonymous substitution in E (V14-, 74% of reads with deletion), N (G>-, 86% of reads), N (A50S)	EPI_ISL_17293256 (PRNT stock sequence)
Omicron BN.1.3.1	T19I, L24S, P25-A27del, H69Y, G142D, K147E, W152R, F157L, I210V, V213G, G257S, G339H,	V78_41249_V.V.p4	Synonymous minor variant (4456, ORF1a, C>T, 10% of reads), (28420, N, T>C, 94% of reads), (28558, N, T>A, 67% of reads), Non-	EPI_ISL_17548230 (PRNT stock sequence)

	R346T, K356T, S371F, S373P, S375F, T376A, D405N, R408S, K417N, N440K, G446S, N460K, S477N, T478K, E484A, F490S, Q498R, N501Y, Y505H, D614G, H655Y, N679K, P681H, N764K, D796Y, Q954H, N969K		synonymous minor variant (10809, C3L proteinase, P252L, 89% of reads), (11750, ORF1a, L3829Y, 8% of reads), (13965, ORF1ab, T4567C, 7% of reads), (14724, ORF1ab, S4820F, 10% of reads), (25337, S, D1259H, 6% of reads), (26261, E, S6L, 6% of reads), (26309, E, A22D, 63% o freads), Non-synonymous substitutions (10809, C3L proteinase, P252L, 89% of reads), (26309, E, A22D, 63% o freads), synonymous substitution (28420, N, T>C, 94% of reads), (28558, N, T>A, 67% of reads)	
Omicron XBB.2	T19I, L24S, P25-A27del, V83A, G142D, Y144-, H146Q, Q183E, V213E, D253G, G339H, R346T, L368I, S371F, S373P, S375F, T376A, D405N, R408S, K417N, N440K, V445P, G446S, N460K, S477N, T478K, E484A, F486S, F490S, Q498R, N501Y, Y505H, D614G, H655Y, N679K, P681H, N764K, D796Y, Q954H, N969K	V79_41245_V.V.p4	Non-synonymous minor variant (9852, ORF1a, D3196A, 39% of reads), (11634, ORF1a, C3790L, 33% of reads), (11635, ORF1a, C3790E, 37% of reads), (12357, ORF1a, T4031None, 6% of reads), (26369, E, Y42C, 12% of reads), (26284-26286, E, deletion, 76% of reads), Synonymous minor variant (27167, M, C>T, 14% of reads) Non-synonymous substitution (26284-26286, E, deletion, 76% of reads)	EPI_ISL_17549547 (PRNT stock sequence)
Omicron EG.5.1	T19I; L24S; P25-A27del; Q52H; V83A; G142D; Y144del; H146Q; Q183E; V213E; G252V; G339H; R346T; L368I; S371F; S373P; S375F; T376A; D405N; R408S; K417N; N440K; V445P; G446S; F456L; N460K; S477N; T478K; E484A; F486P; F490S; Q498R; N501Y; Y505H; D614G; H655Y; N679K; P681H; N764K; D796Y; Q954H; N969K	V140_43769_V.V.p1	Non-synonymous minor variant, S:D839E, 5% of reads	EPI_ISL_18370587 (PRNT stock sequence)
Omicron JN.1	ins17MPLF; T19I; R21T; L24S; P25-A27del; S50L; H69-V70del; V127F; G142D; Y144del; F157S; R158G; N211I; L212-; V213G;	V148_44042_V.V.p1	None	EPI_ISL_18474148

	L216F; H245N; A264D; I332V; G339H; K356T; S371F; S373P; S375F; T376A; R403K; D405N; R408S; K417N; N440K; V445H; G446S; N450D; L452W; L455S; N460K; S477N; T478K; N481K; V483del; E484K; F486P; Q498R; N501Y; Y505H; E554K; A570V; D614G; P621S; H655Y; N679K; P681R; N764K; D796Y; S939F; Q954H; N969K; P1143L			

Table S1: Description of isolates. *Re-sequencing of the stock used for hamster infection showed a non-synonymous substitution in S (S:L179F) for this isolate.

Serum group	Serum	Virus	Adapted titer	Original titer
Alpha sera	2.1	Alpha	3795	3010.13
Alpha sera	2.2	BA.2	640	830.37
Beta sera	3.2	BA.2-12	183.5	<160
Beta sera	3.3	Alpha	968.83	1372.73
Beta sera	3.3	Beta	1824.34	2057.81
Delta sera	4.1	Alpha	5120	>5120
Delta sera	4.2	Delta	3570.23	3125.64
Delta sera	4.3	Alpha	1622.41	2382.62
Delta sera	4.3	Delta	5120	>5120
BA.1 sera	5.1	Alpha	<40	2898.75
BA.1 sera	5.1	BA.2-12	326.59	409.82
BA.1 sera	5.2	Alpha	164.89	205.25
BA.1 sera	5.2	BA.2	225.11	163.89
BA.1 sera	5.3	BA.2	220.56	<160
BA.2-12 sera	6.1	BA.2	5113.91	4699.22
BA.2-12 sera	6.2	BA.2	5330.82	>5120
B.1+E484K sera	7.1	D614G	5442.78	>5120
B.1+E484K sera	7.1	Alpha	5032.42	>5120
B.1+E484K sera	7.1	Delta	2895.08	2116.85
B.1+E484K sera	7.1	B.1+E484K	2635.04	2437.81
B.1+E484K sera	7.1	BA.2-12	2859.64	2607.2
B.1+E484K sera	7.1	Mu	>5120	6364.97
B.1+E484K sera	7.2	Mu	2034.42	2419.48
B.1+E484K sera	7.3	Alpha	4540.72	2962.97
B.1+E484K sera	7.3	Mu	>5120	5237.84

D614G sera	8.1	BA.2-12	320	557.5
BA.5 sera	9.1	BF.7	1647.68	1922.55
BA.5 sera	9.3	BQ.1.18	3539.07	2909.38

Table S2: Adaptations made to the PRNT90 titers inferred when constraining the neutralization curve at zero. In the titrations above, visual inspection of the titer curve revealed that the neutralization curve fixed at zero did not give an ideal fit. Therefore, the adaptations listed above were made to those titers.

Sera	BA.2 sera			XBB.2 sera			Alpha sera		Beta sera			Delta sera			BA.1 sera			BA.2-12 sera			B.1+E484K sera			D614G sera			BA.5 sera		
**Variant**	1.1	1.2	1.3	12SE0030	12SE0031	12SE0032	2.1	2.2	3.1	3.2	3.3	4.1	4.2	4.3	5.1	5.2	5.3	6.1	6.2	6.3	7.1	7.2	7.3	8.1	8.2	8.3	9.1	9.2	9.3
**BF.7**	146.74	118.41	124.73	*	*	*	*	*	*	*	*	*	*	*	84.13	<80	<80	*	*	*	*	*	*	<40	<40	<40	1647.68	903.16	3366.47
**BN.1.3.1**	119.1	<54	<54	*	*	*	*	*	*	*	*	*	*	*	110.94	<54	<54	*	*	*	*	*	*	<40	<40	36.65	124.71	<54	83.58
**BQ.1.18**	<160	<160	<160	*	*	*	*	*	*	*	*	*	*	*	<80	<80	<80	*	*	*	*	*	*	<40	<40	<40	1407.34	628.91	3539.07
**EG.5.1**	<40	42.01	58.07	288.45	553.94	432.03	*	*	*	*	*	*	*	*	170.28	<40	<40	*	*	*	*	*	*	*	*	*	284.31	127.03	191.47
**JN.1**	74.44	<40	<40	129.25	260.74	242.01	*	*	*	*	*	*	*	*	77.72	<40	81.45	*	*	*	*	*	*	*	*	*	153.64	66.88	<40
**Alpha**	81.4	162.57	171.62	*	*	*	3795	5141.54	601.6	1353.24	968.83	5120	1924.14	1622.41	<40	164.89	163.6	171.93	97.49	213.96	5032.42	1462.89	4540.72	1904.11	631.29	2386	*	*	*
**BA.1**	402.62	259.91	549.12	*	*	*	<160	405.82	<160	<160	193.46	422.82	422.82	277.32	1654.9	1658.02	1217.46	1822.46	775.17	806.31	793.69	666.08	622.1	<160	<160	202.45	<160	<160	<160
**BA.2-12**	1407.05	1524.07	1729.68	*	*	*	<160	542.95	232.39	183.5	272.15	959.2	528.57	1043.33	326.59	164.59	200.04	3278.21	4818.17	1299.74	2859.64	903.16	1431	320	<160	275.36	*	*	*
**BA.2**	1855.69	2180.47	1666.72	*	*	*	<160	640	296.38	<160	251.05	702.22	435	711.89	325.46	225.11	220.56	5113.91	5330.82	1287.85	1698.99	652.13	1296.63	512.15	<160	246.5	<160	<160	190.03
**BA.4**	233.96	190.77	366.16	*	*	*	44.62	215.89	238.22	67.78	78.59	533.97	193.77	364.2	187.79	<160	<160	446.83	3156.46	<160	537.69	145.55	271.67	226.61	<20	66.45	*	*	*
**BA.5**	188.53	198.87	214.58	219.94	212.78	159.84	46.78	302.49	190.45	43.74	108.21	558.17	160.75	327.05	258.12	<160	<160	573.32	4027.1	<160	417.36	255.96	198.73	327.89	<20	60.13	1104.83	608	2168.32
**Beta**	<80	238.91	282.05	*	*	*	530.07	1427.84	2237.52	1492.88	1824.34	2304.76	742.18	1032.52	<20	160.28	124.64	437.18	156.13	336.45	2609.58	2052.2	2011.73	1334.84	217.45	1457.76	*	*	*

**Delta**	22.1	<80	<80	*	*	*	383.2	872.03	227.15	<160	190.95	4819.28	3570.23	5120	<20	82.71	55.2	67.59	<40	40.2	2895.08	1375.08	1124	1316.81	502.85	1165.18	*	*	*
**Mu**	<80	322.59	114.34	*	*	*	753.5	1838.21	779.6	546.07	781.76	1771.11	431.69	860.77	<80	86.95	<80	444.51	229.97	561.78	>5120	2034.42	>5120	1213.59	63.97	498.23	*	*	*
**D614G**	<160	<160	<160	<80	<80	<80	889.26	1544.79	402.48	415.96	516.13	1819.79	877.49	1202.49	<160	<160	<160	<160	<160	<160	5442.78	1417.62	2894.1	1472.22	666.78	1503.46	<40	<40	<40
**XBB.2**	70.26	<107	155.4	1765.51	1898.36	885.62	*	*	*	*	*	*	*	*	189.3	<54	<54	*	*	*	*	*	*	<32	<32	<32	342.05	221.84	685.13
**B.1+ E484K**	<80	<80	<80	*	*	*	267.36	393.87	350.87	268.63	286.73	1540.75	329.15	548.17	<40	84.72	54.27	169.35	<80	87.35	2635.04	1426.16	2290.61	693.86	267.98	1576.85	*	*	*

Table S3: Titers used to make the antigenic map. Columns correspond to the different sera, with the infecting variant given in the top row, rows correspond to the variant that was titrated. * indicate variant and serum pairs that have not been titrated.

Dataset S1 (separate file): Raw plaque count data.

Dataset S2 (separate file): Neutralization curves. Each sub-plot shows the neutralization curve for a variant titrated against a serum, as specified in the panel title. The y-axis shows the fraction of infectivity remaining, the x-axis shows the dilution. Blue and orange shapes show the fraction of infectivity remaining from the two runs that were done. Titer curves were fit with the neutcurve package (1). The black curve shows the titer curve inferred when constraining the lower end of the neutralization curve at zero and the upper end at one. The red curve shows the titer curve inferred when constraining the upper end of the neutralization curve at one. The blue curve shows the titer curve inferred when constraining the bottom end of the neutralization curve at 0, and the gray curve shows the titer curve inferred when not constraining the neutralization curve.

References

1. S. H. Wilks, *et al.*, Mapping SARS-CoV-2 antigenic relationships and serological responses. *Science* **382**, eadj0070 (2023).

2. A. Rössler, *et al.*, BA.2 and BA.5 omicron differ immunologically from both BA.1 omicron and pre-omicron variants. *Nat. Commun.* **13**, 7701 (2022).

3. J. Bloom, neutcurve documentation — neutcurve 0.5.7 documentation. GitHub. https://github.com/jbloomlab/neutcurve (accessed 2 September 2022).
